# Phosphorylated SHMT2 Regulates Oncogenesis Through m^6^A Modification in Lung Adenocarcinoma

**DOI:** 10.1002/advs.202307834

**Published:** 2024-03-09

**Authors:** Tianyu Han, Yanan Wang, Minzhang Cheng, Qifan Hu, Xiaorui Wan, Menglin Huang, Yuhan Liu, Wenze Xun, Jin Xu, Lei Wang, Ruiguang Luo, Yi Yuan, Keru Wang, Jianbin Wang

**Affiliations:** ^1^ Jiangxi Institute of Respiratory Disease Department of Respiratory and Critical Care Medicine The First Affiliated Hospital Jiangxi Medical College Nanchang University Nanchang City Jiangxi 330006 China; ^2^ Jiangxi Clinical Research Center for Respiratory Diseases Nanchang City Jiangxi 330006 China; ^3^ China‐Japan Friendship Jiangxi Hospital National Regional Center for Respiratory Medicine Nanchang City Jiangxi 330200 China; ^4^ Department of Thoracic Surgery The First Affiliated Hospital Jiangxi Medical College Nanchang University Nanchang City Jiangxi 330006 China; ^5^ School of Basic Medical Sciences Nanchang University Nanchang City Jiangxi 330031 China; ^6^ School of Huankui Academy Nanchang University Nanchang City Jiangxi 330031 China

**Keywords:** lung adenocarcinoma, N^6^‐methyladenosine, phosphorylation, SHMT2, ubiquitination

## Abstract

Targeting cancer‐specific metabolic processes is a promising therapeutic strategy. Here, this work uses a compound library that directly inhibits metabolic enzymes to screen the potential metabolic targets in lung adenocarcinoma (LUAD). SHIN1, the specific inhibitor of serine hydroxymethyltransferase 1/2 (SHMT1/2), has a highly specific inhibitory effect on LUAD cells, and this effect depends mainly on the overexpression of SHMT2. This work clarifies that mitogen‐activated protein kinase 1 (MAPK1)‐mediated phosphorylation at Ser90 is the key mechanism underlying SHMT2 upregulation in LUAD and that this phosphorylation stabilizes SHMT2 by reducing STIP1 homology and U‐box containing protein 1 (STUB1)‐mediated ubiquitination and degradation. SHMT2‐Ser90 dephosphorylation decreases S‐adenosylmethionine levels in LUAD cells, resulting in reduced N^6^‐methyladenosine (m^6^A) levels in global RNAs without affecting total protein or DNA methylation. Methylated RNA immunoprecipitation sequencing (MeRIP‐Seq) and RNA sequencing (RNA‐Seq) analyses further demonstrate that SHMT2‐Ser90 dephosphorylation accelerates the RNA degradation of oncogenic genes by reducing m^6^A modification, leading to the inhibition of tumorigenesis. Overall, this study elucidates a new regulatory mechanism of SHMT2 during oncogenesis and provides a theoretical basis for targeting SHMT2 as a therapeutic target in LUAD.

## Graphical abstract

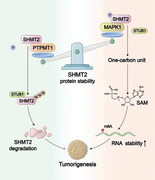

## 1 Introduction

Metabolic reprogramming is one of the hallmarks of cancer.^[^ ^1^ ^]^ Cancer cells obtain a great amount of energy and metabolic intermediates through metabolic reprogramming to synthesize building blocks for rapid proliferation and invasion.^[^ ^2^ ^]^ One‐carbon metabolism contributes to the biosynthesis of macromolecules (proteins, nucleotides, etc.) and functional metabolites (ATP, GSH, NADH, etc.) to maintain cancer progression.^[^ ^3^ ^]^ The major source of one‐carbon units in cancer cells is serine, whose synthesis is catalyzed by serine hydroxymethyltransferase (SHMT) to form glycine and one‐carbon units.^[^ ^4^ ^]^ Both glycine and one‐carbon units can be directly incorporated into the synthesis of nucleotides, proteins, lipids and functional metabolites. In addition, one‐carbon units can also contribute to the methylation of cellular components by supporting the methionine cycle for S‐adenosylmethionine (SAM) production.^[^ ^3^ ^]^ Thus, serine catabolism is necessary for cancer initiation and progression.

Serine hydroxymethyltransferase (SHMT) is the key enzyme involved in serine catabolism.^[^ ^5^ ^]^ SHMT has two isoforms in the human body: SHMT1 is located in the cytoplasm, and SHMT2 is located in the mitochondria.^[^ ^6^ ^]^ There is also a short form of SHMT2, SHMT2α, located in the cytoplasm; this protein mainly functions in the BRISC complex and has no enzymatic activity toward serine.^[^ ^7^, ^8^, ^9^ ^]^ The functions of SHMT1 and SHMT2 may be different in cancer cells. Many studies on one‐carbon fluxes have shown that SHMT1 catalyzes the reaction toward serine synthesis, while SHMT2 catalyzes serine catabolism.^[^ ^10^ ^]^ Previously, both SHMT1 and SHMT2 were demonstrated to play important roles in cancer. Studies have shown that the expression of SHMT2 is upregulated in many types of cancer, including breast cancer, liver cancer, gastric cancer, and colon cancer. SHMT2 overexpression promotes cancer cell proliferation, invasion and tumorigenesis, and knocking down SHMT2 inhibits tumor progression.^[^ ^11^, ^12^, ^13^, ^14^ ^]^ SHMT2‐mediated mitochondrial serine metabolism was also reported to drive the drug resistance of cancer cells.^[^ ^15^ ^]^ SHMT2 regulates the methylation of mitochondrial tRNA and mediates the translation of respiratory chain proteins by providing one‐carbon units.^[^ ^16^, ^17^ ^]^ SHMT2 can also initiate lymphoma development by regulating DNA methylation and silencing tumor suppressors.^[^ ^18^ ^]^ As for SHMT1, some studies have shown that SHMT1 is upregulated in cancers such as lung cancer and low‐grade glioma, and SHMT1 knockdown inhibits the proliferation of cancer cells.^[^ ^19^, ^20^ ^]^ However, in hepatocellular carcinoma, SHMT1 was downregulated and functioned as a tumor suppressor.^[^ ^21^ ^]^ Although SHMT1 is also a serine catabolic protein, a study reported that SHMT1 expression did not significantly affect methylation capacity.^[^ ^22^ ^]^ Thus, clarifying the specific functions of the two isoforms in a certain type of cancer is important for understanding cancer progression.

Because of the importance of serine catabolism in cancer, elucidating the regulatory mechanisms of SHMT is a hot topic in cancer metabolism research. NFE2 like bZIP transcription factor 2 (NRF2) was demonstrated to regulate the transcription of serine metabolic genes, including SHMT1/2, through activating transcription factor 4 (ATF4) to support glutathione and nucleotide production.^[^ ^23^ ^]^ Under nutrient deprivation conditions, cMyc can also transcriptionally upregulate the expression of SHMT1/2 to promote the survival and proliferation of cancer cells.^[^ ^24^ ^]^ Nuclear enrichment of glycogen synthase kinase 3 (GSK3) can suppress the transcription and expression of SHMT2.^[^ ^25^ ^]^ In addition to transcriptional regulation, insulin like growth factor 2 mRNA binding protein 1 (IGF2BP1) was demonstrated to increase SHMT2 expression by stabilizing its mRNA.^[^ ^26^ ^]^ Posttranslational modification is another key mechanism for regulating the function of metabolic enzymes. However, only a small portion of related studies have focused on this topic. Acetylation of Lys95 in SHMT2 disrupted its tetramer structure and inhibited its enzymatic activity. In addition, this acetylation promoted SHMT2 degradation through the ubiquitin‐lysosome pathway.^[^ ^14^ ^]^ In another study, sirtuin 5 (SIRT5)‐mediated desuccinylation of SHMT2 increased its activity and drove serine catabolism in cancer cells.^[^ ^27^ ^]^ SHMT2 was also reported to be a fatty‐acylated protein, and histone deacetylase 11 (HDAC11) mediated its defatty‐acylation.^[^ ^28^ ^]^ Until now, little has been known about the functions of other posttranslational modifications in regulating SHMT in cancer, especially in lung cancer.

In this study, we found that the proliferation of lung adenocarcinoma cells was more strongly dependent on SHMT2 than on SHMT1. The upregulation of SHMT2 in cancer cells was mainly caused by enhanced protein stability, and phosphorylation played a key role in this process. We elucidated that Ser90 of SHMT2 was the key phosphorylation site for regulating its protein stability and that MAPK1/PTPMT1 (protein tyrosine phosphatase mitochondrial 1) was the kinase/phosphotase responsible for this phosphorylation. We also found that STUB1 was an E3 ligase that mediated the proteasomal degradation of dephosphorylated SHMT2. Inhibiting the phosphorylation of SHMT2‐Ser90 inhibited tumorigenesis and reprogrammed cell metabolism. We further discovered that inhibiting this phosphorylation did not affect global DNA or protein methylation but rather decreased global m^6^A modification, leading to the instability of mRNAs and blockage of oncogenic pathways. Thus, this study reveals a new regulatory mechanism for SHMT2 regulation in lung adenocarcinoma and lays a theoretical basis for targeting SHMT2 as a therapeutic target in lung adenocarcinoma.

## 2 Results

### 2.1 SHMT2 is a Potential Therapeutic Target for Lung Adenocarcinoma

**Figure 1:**
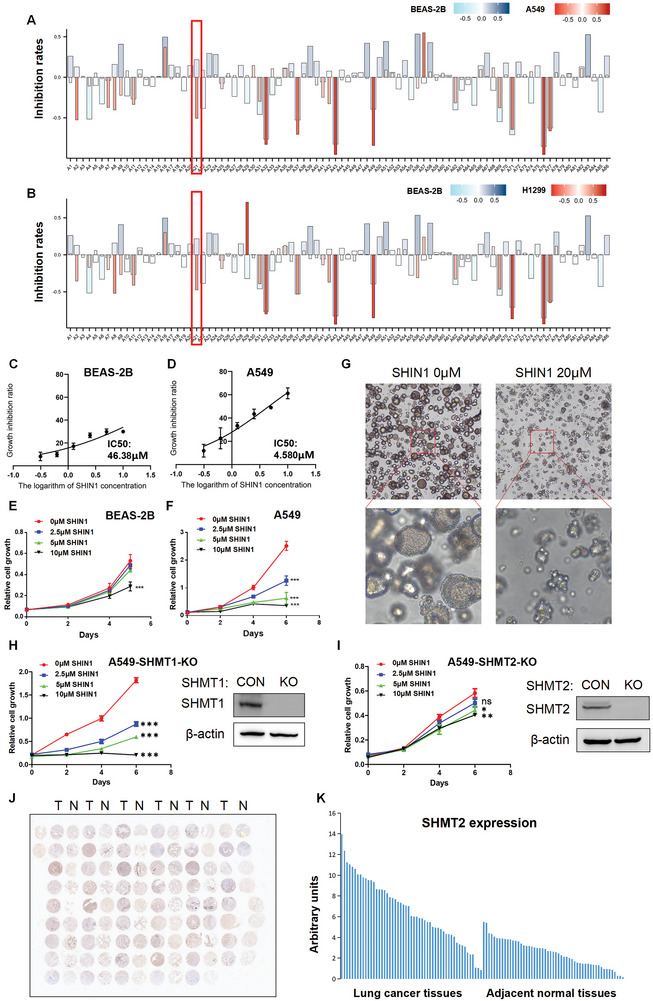
SHMT2 is a potential therapeutic target in lung adenocarcinoma. A,B) A screening compound library directly inhibiting metabolic enzymes was used to treat human bronchial epithelial cells (BEAS‐2B) and LUAD cells (A549 and H1299) at a final concentration of 10 µm. After 48 h, cell viability was determined using a Cell Counting Kit‐8 (CCK8) assay. The inhibition rates were used to plot the images. Positive values indicate growth promotion, and negative values represent growth suppression. Data represent the average of two replications. C,D) Different concentrations of SHIN1 were used to treat BEAS‐2B (C) and A549 cells (D) for 48 h. The growth inhibition rates were measured using an MTT assay. The data represent the average of three independent experiments (mean ± SD). E,F) Different concentrations of SHIN1 were used to treat BEAS‐2B (E) and A549 cells (F) for the indicated times. Cell proliferation was detected using crystal violet staining. The data represent the average of three independent experiments (mean ± SD). ***, *p* < 0.001. G) Patient‐derived lung adenocarcinoma organoids were treated with 20 µm SHIN for 7 days. The images were taken at 40× and 100× magnification. H,I) Different concentrations of SHIN1 were used to treat A549 cells with SHMT1 knockout (A549‐SHMT1‐KO) (H) or SHMT2 knockout (A549‐SHMT2‐KO) (I) for the indicated times. Cell proliferation was detected using crystal violet staining. The data represent the average of three independent experiments (mean ± SD). ns, *p* > 0.05; *, *p* < 0.05; **, *p* < 0.01; ***, *p* < 0.001 (left panels). Western blotting was used to detect protein expression using the indicated antibodies (right panels). J) Microscopic evaluation of immunohistochemistry (IHC) staining of the LUAD tissue microarray with an anti‐SHMT2 antibody. N: adjacent normal tissue; T: tumor tissue. K) Quantification of the IHC staining shown in (J).

Targeting cancer metabolic reprogramming is a promising strategy for cancer treatment.^[^ ^2^ ^]^ To discover effective therapeutic targets in lung adenocarcinoma, we used a screening compound library that directly inhibited metabolic enzymes involved in glycolysis, the tricarboxylic acid cycle (TCA cycle), amino acid metabolism, lipid metabolism, etc., to identify potential targets (Table S1, Supporting Information). **Figure** **1**A,B shows that there were several effective compounds that significantly inhibited the viability of lung adenocarcinoma cells (A549 and H1299) but had minor effects on human bronchial epithelial cells (BEAS‐2B). SHIN1, a specific inhibitor of SHMT, is one of the most effective inhibitors. We next performed drug sensitivity experiments to detect the sensitivity of human bronchial epithelial cells (BEAS‐2B) and lung adenocarcinoma cells to SHIN1. The lung adenocarcinoma cells (A549, H1299, and PC9) had greater sensitivity to SHIN1 (IC50 < 10 µm) than the BEAS‐2B cells (IC50 = 46.38 µm) (Figure 1C,D, Figure S1A,B, Supporting Information). Cell proliferation assays also showed that SHIN1 inhibited the proliferation of lung adenocarcinoma cells at a concentration of 2.5 µm, while similar inhibitory effects needed to reach 10 µm for BEAS‐2B cells (Figure 1E,F, Figure S1C,D, Supporting Information). We subsequently constructed patient‐derived organoids to examine the inhibitory effects of SHIN1. SHIN1 treatment significantly blocked the growth of the lung adenocarcinoma organoids (Figure 1G and Figure S1E, Supporting Information). SHMT has two isoforms: cytoplasmic SHMT1 and mitochondrial SHMT2. Both isoforms are inhibited by SHIN1.^[^ ^29^ ^]^ To determine the role of the two isoforms in the growth inhibition of SHIN1, we constructed stable cell lines in which SHMT1 or SHMT2 was knocked out. Figure S1F,G, Supporting Information shows that both SHMT1 and SHMT2 knockout inhibited the proliferation of LUAD cells. However, SHMT2 knockout had a much greater inhibitory effect than SHMT1 knockout. SHIN1 treatment inhibited the proliferation of A549 cells with SHMT1 knockout (Figure 1H). However, SHMT2 knockout abolished the inhibitory effect of SHIN1 on cell proliferation (Figure 1I). These findings indicated that SHMT2 plays a main role in lung adenocarcinoma cells. To further confirm these results, we knocked down SHMT2 using small interfering RNAs (siRNAs) and examined its effects on cell proliferation. Knocking down SHMT2 significantly blocked the proliferation of LUAD cells, while the proliferation of human bronchial epithelial cells (BEAS‐2B) was not affected, indicating that SHMT2 might be a cancer‐specific target in LUAD (Figure S1G,H, Supporting Information). We measured the protein expression of SHMT1 and SHMT2 in lung adenocarcinoma cells and human bronchial epithelial cells (BEAS‐2B). SHMT2 was more highly expressed in lung adenocarcinoma cells than in human bronchial epithelial cells, while SHMT1 did not show this trend (Figure S1K,L, Supporting Information). We further measured the expression of SHMT2 in a lung adenocarcinoma tissue microarray. The results showed that SHMT2 expression was much higher in cancer tissues than in adjacent normal tissues (Figure 1J,K). These results demonstrate that SHMT2 is a potential therapeutic target in lung adenocarcinoma.

### 2.2 The Protein Stability of SHMT2 is Regulated by Phosphorylation at Ser90

Previous studies demonstrated that the expression of SHMT2 was regulated by several transcription factors, such as ATF4, NRF2 and c‐Myc.^[^ ^23^, ^24^ ^]^ To determine the regulatory mechanisms involved in SHMT2 upregulation in LUAD, we first evaluated the difference in the transcription level of SHMT2 between LUAD tissues and normal tissues using the Gene Expression Profiling Interactive Analysis (GEPIA) platform. We found that the transcription level of SHMT2 in LUAD tissues was slightly increased but was not significantly upregulated (Figure S2A, Supporting Information). We next measured the mRNA expression of SHMT2 in BEAS‐2B and LUAD cells. Figure S2B, Supporting Information shows that the mRNA expression of SHMT2 in some LUAD cells was upregulated. However, SHMT2 mRNA was downregulated in some LUAD cells. These results were inconsistent with the protein expression of SHMT2 in LUAD cells and tissues. As the expression of a protein is regulated by the balance between synthesis and degradation, we next measured the protein degradation rates of SHMT2 in human bronchial epithelial cells and LUAD cells. Cycloheximide (CHX) was used to block protein synthesis, and the results showed that the SHMT2 protein in human bronchial epithelial cells (BEAS‐2B) degraded much faster than that in LUAD cells (Figure S2C–H, Supporting Information), indicating that the upregulation of the SHMT2 protein in LUAD cells was regulated by enhanced protein stability.

**Figure 2:**
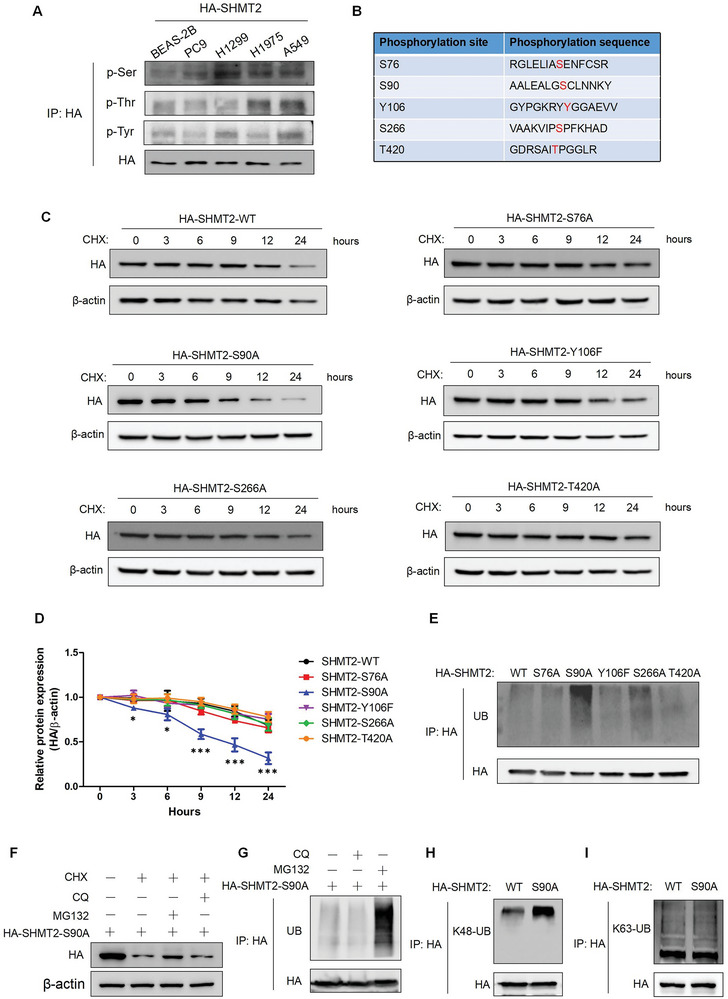
Phosphorylation of SHMT2 at Ser90 regulates its protein stability. A) Human bronchial epithelial cells (BEAS‐2B) and LUAD cells (PC9, H1299, H1975, A549) were transfected with pcDNA3.1‐HA‐SHMT2. 48 h later, immunoprecipitation was performed, and phosphorylation of serine, threonine and tyrosine was detected using the indicated antibodies. B) The phosphorylation sites identified by mass spectrometry. C) Wild‐type HA‐SHMT2 (HA‐SHMT2‐WT) and the corresponding phosphorylation mutants (S76A, S90A, Y106F, S266A, and T420A) were transfected into A549 cells. CHX was used to treat the cells for the indicated times, and protein degradation was detected via western blotting. D) The relative HA‐SHMT2 expression compared with that of β‐actin in (C) was quantified using image J. The relative expression levels of SHMT2 in all cancer cell lines were compared to that in BEAS‐2B cells. Data represent the average of three independent experiments (mean ± SD). *, *p* < 0.05; ***, *p* < 0.001. E) Wild‐type SHMT2 and phosphorylated SHMT2 mutants (S76A, S90A, Y106F, S266A, and T420A) were transfected into A549 cells. 48 h later, immunoprecipitation was performed, and ubiquitination was detected. F) A549 cells transfected with SHMT2‐S90A were treated with CHX only, CHX and MG132, CHX and CQ. The protein expression was detected using western blotting. G) A549 cells transfected with HA‐SHMT2‐S90A were treated with MG132 or CQ. The ubiquitination of SHMT2 was detected using western blotting. H,I) HA‐SHMT2‐WT and HA‐SHMT2‐S90A were transfected into A549 cells, which were subsequently immunoprecipitated using an anti‐HA antibody. Western blotting was used to detect the K48‐linked (H) and K63‐linked (I) ubiquitination of SHMT2.

Phosphorylation is a key posttranslational modification that regulates nearly every aspect of a protein. Our previous study demonstrated that phosphorylation is a key factor for regulating glutaminolysis in lung cancer cells.^[^ ^30^ ^]^ However, little is known about the role of phosphorylation in serine catabolism. To clarify the function of phosphorylation in serine catabolism, we first examined whether phosphorylation occurs in SHMT2 in LUAD cells. **Figure** **2**A shows that phosphorylation of SHMT2 can occur at serine, threonine and tyrosine residues, but only the phosphorylation level of serine increased in LUAD cells compared with that in human bronchial epithelial cells (BEAS‐2B). Considering the faster rate of SHMT2 protein degradation in BEAS‐2B cells than in LUAD cells, we speculated that serine phosphorylation might be associated with the degradation of the SHMT2 protein. To further confirm this result, we used mass spectrometry (MS) to identify the phosphorylation sites of SHMT2 in A549 cells (Table S2, Supporting Information). Five phosphorylation sites (S76, S90, Y106, S266, and T420) were discovered, and point mutations were made (Figure 2B). We then investigated whether phosphorylation affects the protein stability of SHMT2 in LUAD. The stability of these SHMT2 mutants was detected, and the results showed that only the mutation at Ser90 accelerated the degradation of SHMT2 (Figure 2C,D). The ubiquitination of these mutants was subsequently examined. Only SHMT2‐S90A exhibited a marked increase in the ubiquitination of SHMT2 (Figure 2D). However, this mutation did not affect the enzymatic activity of SHMT2 (Figure S2I, Supporting Information). On the contrary, the phosphorylation mimic mutation SHMT2‐S90D showed decreased ubiquitination of SHMT2 and the protein stability was significantly enhanced (Figure S2J,K, Supporting Information). We subsequently examined the degradation pathway of this mutant. The proteasome inhibitor MG132, but not the lysosome inhibitor chloroquine (CQ), reversed the reduced protein expression of SHMT2‐S90A (Figure 2F). Additionally, the ubiquitination of SHMT2‐S90A was significantly upregulated when the cells were treated with MG132 (Figure 2G), indicating that SHMT2‐S90A was degraded through the proteasome pathway. We further explored the ubiquitin chain types of SHMT2‐S90A using specific antibodies. Figure 2H,I shows that the increase in ubiquitination of SHMT2‐S90A was caused by K48 linkage.

We next prepared an antibody specifically targeting SHMT2‐Ser90 phosphorylation. Figure S3A, Supporting Information shows that this antibody could recognize phosphorylated SHMT2‐Ser90. We used this antibody to measure the level of SHMT2‐Ser90 phosphorylation in LUAD cells and human bronchial epithelial cells using total SHMT2 as the loading control. The phosphorylation of SHMT2‐Ser90 was markedly higher in LUAD cells than in human bronchial epithelial cells (Figure S3B, Supporting Information). We then performed IHC staining to detect SHMT2‐Ser90 phosphorylation using LUAD tissue microarray from the same batch in Figure 1J (Figure S3C, Supporting Information). Figure S3C,D, Supporting Information showed that the phosphorylation of SHMT2‐Ser90 was higher in cancer tissues than in adjacent normal tissues. The representative images of IHC results from the same tumor tissues stained with SHMT2 and p‐SHMT2 antibodies were shown in Figure S3E, Supporting Information. We could see that as the expression of phosphorylated SHMT2 increased, the expression of total SHMT2 levels were also upregulated (Figure S3E, Supporting Information). We further used the staining intensity of all the tumor tissues in tissue microarrays in Figure 1J and Figure S3C, Supporting Information to plot the image and evaluated the relationship between the expression of total and phosphorylated SHMT2. Figure S3F, Supporting Information showed that the expression of total and phosphorylated SHMT2 displayed a similar trend. These results suggest that Ser90 is the key phosphorylation site that regulates the ubiquitination and protein stability of SHMT2.

### 2.3 STUB1 Regulates the Ubiquitination and Stability of SHMT2 Phosphorylated at Ser90

**Figure 3:**
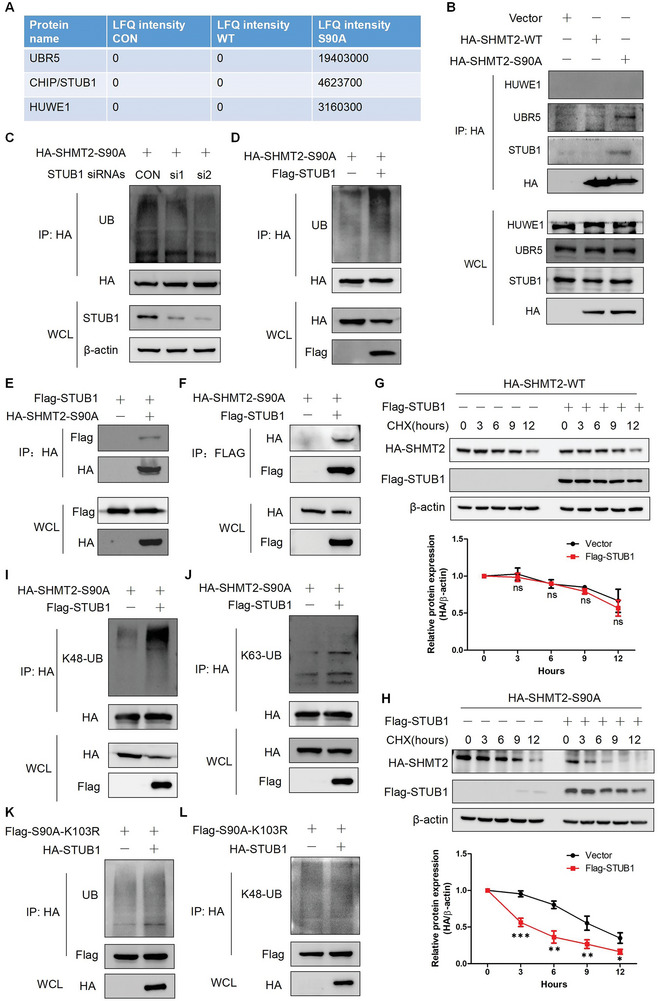
STUB1 ubiquitinates and degrades dephosphorylated SHMT2‐Ser90. A) The E3 ligases that specifically target SHMT2‐S90A identified by mass spectrometry. B) HA‐SHMT2‐WT and HA‐SHMT2‐S90A were transfected into A549 cells, and immunoprecipitation was used to examine the protein interactions. C) A549 cells were cotransfected with the HA‐SHMT2‐S90A plasmid and negative control siRNA or with HA‐SHMT2‐S90A and STUB1‐specific siRNAs. 48 h later, the ubiquitination of SHMT2‐S90A was detected via western blotting. D) A549 cells were cotransfected with the HA‐SHMT2‐S90A plasmid and the Flag‐STUB1 plasmid. 48 h later, the ubiquitination of SHMT2‐S90A was detected via western blotting. E,F) HA‐SHMT2‐S90A and Flag‐STUB1 plasmids were transfected into 293T cells, which were subsequently immunoprecipitated using anti‐HA (E) or anti‐Flag (F) antibodies. Western blotting was used to evaluate protein interactions. G,H) HA‐SHMT2‐WT (G) and HA‐SHMT2‐S90A (H) plasmids were transfected with or without the Flag‐STUB1 plasmid into A549 cells. CHX was used to treat the cells for the indicated times, and protein degradation was detected via western blotting (Top panels). The relative HA‐SHMT2 expression level compared with that of β‐actin was quantified. The data represent the average of three independent experiments (mean ± SD). ns, *p* > 0.05; *, *p* < 0.05; **, *p* < 0.01; ***, *p* < 0.001 (Bottom panels). I,J) HA‐SHMT2‐S90A plasmids with or without the Flag‐STUB1 plasmid were transfected into A549 cells, which were subsequently immunoprecipitated using an anti‐HA antibody. Western blotting was used to detect K48‐linked (I) and K63‐linked (J) ubiquitination. K,L) Flag‐SHMT2‐S90A‐K103R with or without the HA‐STUB1 plasmid was transfected into A549 cells, which were immunoprecipitated using an anti‐Flag antibody. Western blotting was used to detect total (K) and K48‐linked (L) ubiquitination.

We next investigated the mechanism underlying the rapid degradation of the SHMT2‐S90A protein. Mass spectrometry was used to identify the E3 ligases that specifically interact with SHMT2‐S90A (Table S3, Supporting Information). Three E3 ligases: ubiquitin protein ligase E3 component n‐recognin 5 (UBR5), STUB1, and HECT, UBA and WWE domain containing E3 ubiquitin protein ligase 1 (HUWE1) were identified among the immunoprecipitates enriched with SHMT2‐S90A but not SHMT2‐WT (**Figure** **3**A). We confirmed this result using immunoprecipitation. Figure 3B shows that UBR5 and STUB1 could bind to SHMT2‐S90A and that HUWE1 did not interact with these proteins. We then knocked down UBR5 and STUB1 using specific siRNAs and examined the ubiquitination of SHMT2. Knocking down STUB1, but not UBR5, significantly decreased the ubiquitination of SHMT2‐S90A (Figure 3C and Figure S4A, Supporting Information). Overexpressing STUB1 increased the ubiquitination of SHMT2‐S90A (Figure 3D). These results indicated that STUB1 might be an E3 ligase regulating the ubiquitination and stability of SHMT2‐S90A. We confirmed the interaction between STUB1 and SHMT2‐S90A. Figure 3E,F shows that STUB1 and SHMT2‐S90A interact with each other. The effects of STUB1 expression on the degradation of SHMT2 and SHMT2‐S90A were detected. Overexpressing STUB1 significantly accelerated the degradation of SHMT2‐S90A, while no significant effects were observed on the degradation of SHMT2‐WT (Figure 3G,H). We also constructed an A549 stable cell line in which Ser90 of endogenous SHMT2 was mutated to an alanine residue (A549‐S90A) (Figure S4B, Supporting Information). Overexpressing STUB1 significantly accelerated the degradation rate of SHMT2 in A549‐S90A cells but not in wild‐type A549 cells (A549‐WT) (Figure S4C,D, Supporting Information). The inactive mutant STUB1‐H260Q was also used to detect its effects on SHMT2‐S90A ubiquitination.^[^ ^31^ ^]^ Figure S4E, Supporting Information shows that wild‐type STUB1 could increase the ubiquitination of SHMT2‐S90A, but STUB1‐H260Q did not have this effect. We further explored the regulatory effects of STUB1 on the ubiquitin chain types of SHMT2‐S90A using specific antibodies. Figure 3I,J shows that STUB1 ubiquitinated SHMT2‐S90A of the K48‐linkage. These results were further confirmed using ubiquitin mutants (K63R, Lys63 was mutated to arginine; K48R, Lys48 was mutated to arginine). STUB1 still increased SHMT2‐S90A ubiquitination when transfected with Ub‐K63R, while transfecting Ub‐K48R abolished the increased ubiquitination of SHMT2‐S90A (Figure S4F,G, Supporting Information). These results demonstrated that STUB1 regulates the ubiquitination and stability of SHMT2‐S90A.

We then intended to identify the key ubiquitination site on SHMT2‐S90A regulated by STUB1. The PhosphoSitePlus platform was used to identify potential ubiquitination sites in SHMT2. Fourteen ubiquitination sites were found, and point mutations were performed to mutate these lysine residues to arginine residues in the SHMT2‐S90A plasmid (Figure S5A, Supporting Information). Next, we investigated the effects of STUB1 overexpression on the ubiquitination of these mutants. Mutation of Lys103 abolished the increase in ubiquitination of SHMT2‐S90A when STUB1 was overexpressed, while STUB1 still promoted the ubiquitination of other mutants (Figure 3K,L, S5B–S5N). Mutation of Lys103 also slowed the protein degradation rate of SHMT2‐S90A (Figure S6A, Supporting Information), and overexpressing STUB1 did not increase the degradation rate of SHMT2‐S90A/K103R (Figure S6B, Supporting Information). These results showed that Lys103 was the key ubiquitination site in SHMT2‐S90A regulated by STUB1.

### 2.4 MAPK1 Inhibition Reduces the Protein Stability of SHMT2

**Figure 4:**
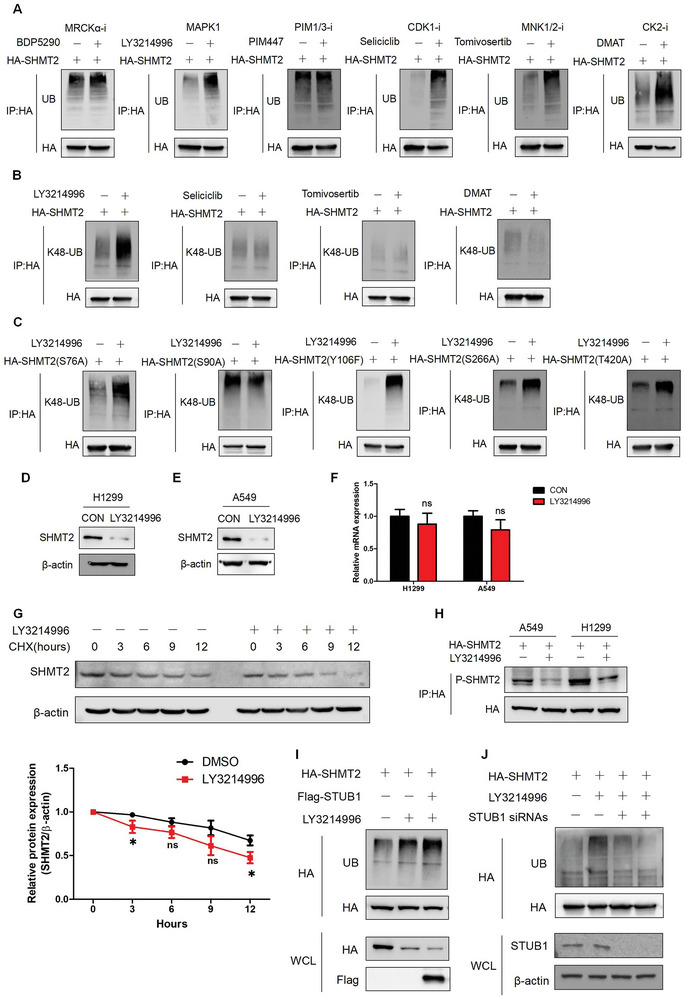
MAPK1 inhibition reduces the protein stability of SHMT2. A) A549 cells transfected with HA‐SHMT2 were separately treated with 1 µm BDP5290, 1 µm LY3214996, 1 µm PIM447, 10 µm seliciclib, 1 µm tomivosertib, and 10 µm DMAT for 24 h. The ubiquitination of SHMT2 was detected using western blotting. B) A549 cells transfected with HA‐SHMT2 were separately treated with 1 µm LY3214996, 10 µm seliciclib, 1 µm tomivosertib, or 10 µm DMAT. K48‐linked ubiquitination of SHMT2 was detected using western blotting. C) A549 cells transfected with the SHMT2 mutants were treated with 1 µm LY3214996. K48‐linked ubiquitination of SHMT2 was detected using western blotting. D,E) H1299 (D) and A549 (E) cells were treated with 1 µm LY3214996 for 24 h. Western blotting was used to detect protein expression. F) H1299 and A549 cells were treated with 1 µm LY3214996 for 24 h. Q‐PCR was used to detect mRNA expression. The data represent the average of three independent experiments (mean ± SD). ns, *p* > 0.05. G) A549 cells were treated with CHX alone or with CHX or LY3214996 for the indicated times. Protein degradation was detected using western blotting (Top panel). The relative SHMT2 expression compared with that of β‐actin was quantified. The data represent the average of three independent experiments (mean ± SD). ns, *p* > 0.05; *, *p* < 0.05 (Bottom panels). H) A549 and H1299 cells transfected with HA‐SHMT2 were treated with 1 µm LY3214996. Immunoprecipitation was used to concentrate HA‐SHMT2. The phosphorylation of SHMT2‐Ser90 was detected using an antibody specifically targeting SHMT2‐Ser90. I) A549 cells were transfected with the indicated plasmids and treated with or without LY3214996. Western blotting was used to detect protein ubiquitination. J) A549 cells were transfected with the indicated plasmid and siRNAs and then treated with or without LY3214996. Western blotting was used to detect protein ubiquitination.

As phosphorylation at Ser90 regulates the stability of SHMT2, another important question is how to elucidate the regulatory mechanism of SHMT2‐Ser90 phosphorylation. According to our mass spectrometry results, three kinases MAPK1, cyclin dependent kinase 1 (CDK1) and casein kinase 2 (CK2) interact with SHMT2. We also used GPS5.0 to identify potential kinases, and CDC42 binding protein kinase alpha (MRCKα), serine/threonine‐protein kinase pim‐1/3 (PIM1/3) and MAPK interacting serine/threonine kinase 1/2 (MNK1/2) were identified. We used specific inhibitors targeting these kinases to treat cells, and the ubiquitination of SHMT2 was detected. **Figure** **4**A shows that inhibiting MAPK1, CDK1, MNK1/2 and CK2 increased the ubiquitination of SHMT2. However, only MAPK1 inhibition (LY3214996) increased the K48‐linked ubiquitination of SHMT2 (Figure 4B). The effects of LY3214996 on the ubiquitination of SHMT2 mutants were also examined. LY3214996 treatment increased the ubiquitination of all the SHMT2 mutants except for S90A (Figure 4C). These results suggested that the phosphorylation at Ser90 might be regulated by MAPK1. The effects of LY3214996 on SHMT2 expression were subsequently investigated. LY3214996 treatment significantly reduced the protein expression of SHMT2 in LUAD cells but did not significantly influence the mRNA level of SHMT2 (Figure 4D–F). LY3214996 treatment also accelerated the degradation of SHMT2 in A549 cells (Figure 4G). We then detected the effects of LY3214996 on SHMT2 phosphorylation. As shown in Figure 4H, LY3214996 treatment significantly decreased the phosphorylation of SHMT2‐Ser90. As STUB1 regulates the degradation of SHMT2 at Ser90, we next detected the effects of STUB1 expression on the ubiquitination of SHMT2 in cells treated with LY3214996. Figure 4I,J shows that overexpressing STUB1 enhanced LY3214996‐mediated ubiquitination of SHMT2, while knocking down STUB1 abolished the SHMT2 ubiquitination induced by LY3214996 treatment. These results indicate that MAPK1 is a potential kinase regulating the phosphorylation and protein stability of SHMT2 in LUAD.

### 2.5 MAPK1 Regulates the Phosphorylation of SHMT2‐Ser90

**Figure 5:**
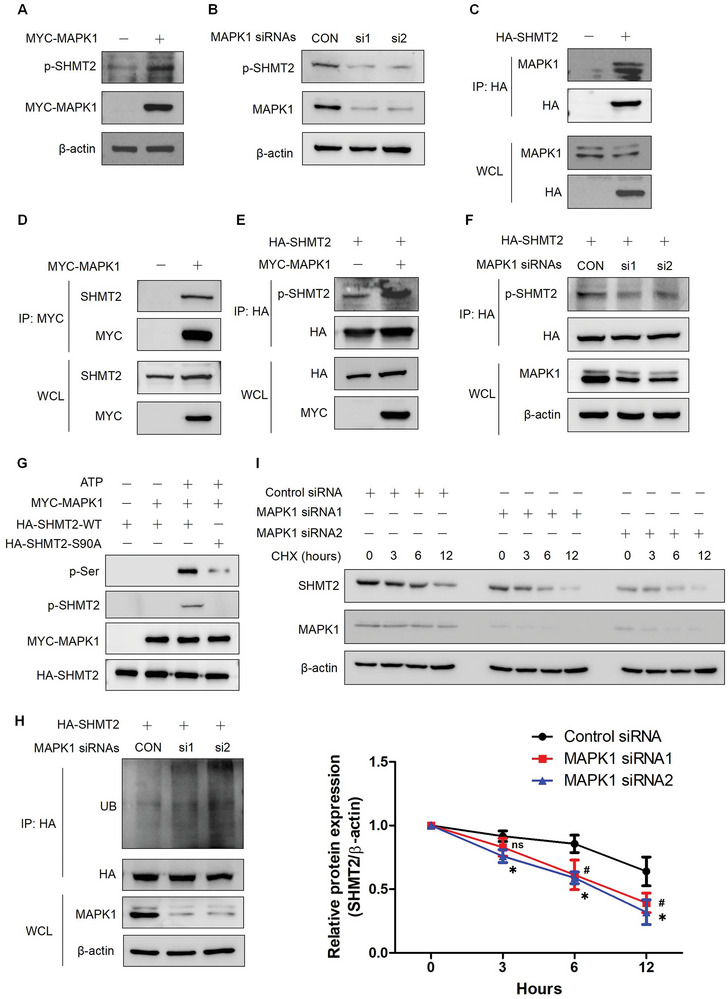
MAPK1 regulates the phosphorylation of SHMT2‐Ser90. A) A549 cells were transfected with MYC‐MAPK1. Western blotting was used to detect protein expression. B) A549 cells were transfected with MAPK1 siRNAs. Western blotting was used to detect protein expression. C) A549 cells were transfected with HA‐SHMT2. Immunoprecipitation was used to concentrate HA‐SHMT2. Western blotting was used to detect protein expression. D) A549 cells were transfected with MYC‐MAPK1. Immunoprecipitation was used to concentrate MYC‐MAPK1. Western blotting was used to detect protein expression. E) A549 cells were transfected with the indicated plasmids. Immunoprecipitation was used to concentrate HA‐SHMT2. Western blotting was used to detect protein expression. F) A549 cells were transfected with HA‐SHMT2 and MAPK1 siRNAs. Immunoprecipitation was used to concentrate HA‐SHMT2. Western blotting was used to detect protein expression. G) HA‐SHMT2 was concentrated from BEAS‐2B cells, and MYC‐MAPK1 was concentrated from A549 cells for use in an in vitro kinase assay. Western blotting was used to detect protein expression. H) A549 cells were transfected with HA‐SHMT2 and MAPK1 siRNAs. Immunoprecipitation was used to concentrate HA‐SHMT2, and western blotting was used to detect protein expression. I) A549 cells were transfected with control or MAPK1 siRNAs. The cells were treated with CHX for the indicated times, and western blotting was used to detect protein expression (Top panel). The relative SHMT2 expression compared with that of β‐actin was quantified. The data represent the average of three independent experiments (mean ± SD). #, siRNA1 versus Control; *,siRNA2 versus Control. ns, *p* > 0.05; *, *p* < 0.05; #, *p* < 0.05 (Bottom panels).

We next examined whether MAPK1 directly regulates the phosphorylation of SHMT2‐Ser90. As SHMT2 is a mitochondrial enzyme, we first detected whether MAPK1 also located in mitochondria in LUAD cells. Through mitochondrial isolation experiments, we discovered that MAPK1 distributed in both cytoplasm and mitochondria, and the phosphorylated SHMT2 resided nearly exclusively in mitochondria (Figure S7A, Supporting Information). Overexpression of MAPK1 increased the phosphorylation of SHMT2 at Ser90, while knocking down MAPK1 reduced this phosphorylation (**Figure** **5**A,B). We also demonstrated that MAPK1 interacted with SHMT2, and the N‐terminal of SHMT2 (1–204aa) is critical for this interaction (Figure 5C,D and Figure S7B, Supporting Information). To further confirm that MAPK1 regulates SHMT2 phosphorylation, SHMT2 was immunoprecipitated, and its phosphorylation at Ser90 was measured. Figure 5E,F shows that overexpressing MAPK1 significantly increased SHMT2 phosphorylation, while MAPK1 knockdown had the opposite effect. We then performed an in vitro kinase assay. The results demonstrated that MAPK1 phosphorylated SHMT2 at Ser90 (Figure 5G). We also found that MAPK1 could phosphorylate SHMT2‐S90A on other serine residues, although this phosphorylation level was much lower than that of SHMT2‐WT. This indicated that there were other SHMT2 phosphorylation sites exist except for Ser90 which were regulated by MAPK1. Then, the ubiquitination of SHMT2 was detected in cells with MAPK1 knockdown. Figure 5H shows that knocking down MAPK1 increased the ubiquitination of SHMT2. We also investigated the effects of MAPK1 on the stability of SHMT2. Knocking down MAPK1 accelerated the degradation rate of SHMT2 (Figure 5I). Finally, we examined the effects of MAPK1 inhibition on the proliferation of LUAD cells. Different concentrations of the MAPK1 inhibitor‐LY3214996 were used to treat LUAD cells and cell proliferation assay was performed. Figure S7C,D, Supporting Information showed that LY3214996 treatment significantly inhibited the proliferation of LUAD cells even at a low concentration (1 µm). We also knocked down MAPK1 using specific siRNAs followed by cell proliferation assay. Knocking down MAPK1 remarkably attenuated the proliferation of LUAD cells (Figure S7E,F, Supporting Information). All these results demonstrated that MAPK1 regulates the phosphorylation of SHMT2‐Ser90 and the protein stability of SHMT2.

### 2.6 PTPMT1 Dephosphorylates SHMT2 at Ser90 and Regulates the Protein Stability of SHMT2

**Figure 6:**
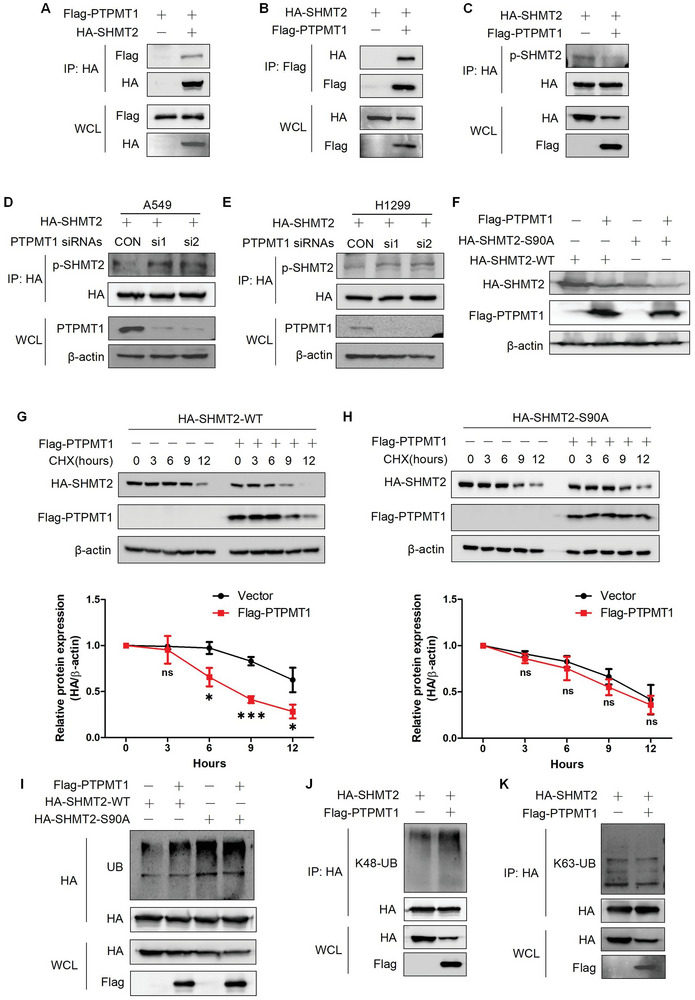
PTPMT1 dephosphorylates SHMT2 at Ser90 and regulates the protein stability of SHMT2. A,B) 293T cells were treated with the indicated plasmids. Immunoprecipitation combined with western blotting was used to detect protein interactions. C) A549 cells were transfected with the indicated plasmids. Immunoprecipitation was used to concentrate HA‐SHMT2. Western blotting was used to detect protein expression. D,E) A549 (D) and H1299 (E) cells were transfected with HA‐SHMT2 and PTPMT1 siRNAs. Immunoprecipitation was used to concentrate HA‐SHMT2, and western blotting was used to detect protein expression. F) A549 cells were transfected with the indicated plasmids. Western blotting was used to detect protein expression. G,H) A549 cells transfected with SHMT2‐WT (G) or SHMT2‐S90A (H) were cotransfected with Flag‐PTPMT1. The cells were treated with CHX for the indicated times, and western blotting was used to detect protein expression (Top panels). The relative HA‐SHMT2 expression level compared with that of β‐actin was quantified. The data represent the average of three independent experiments (mean ± SD). ns, *p* > 0.05; *, *p* < 0.05; ***, *p* < 0.001 (Bottom panels). I) A549 cells were transfected with the indicated plasmids. Immunoprecipitation combined with western blotting was used to detect protein expression. J,K) A549 cells were transfected with the indicated plasmids. Immunoprecipitation was used to concentrate HA‐SHMT2. Western blotting was used to detect K48‐linked (J) and K63‐linked (K) ubiquitination.

PTPMT1 is a protein phosphatase that is located mainly in mitochondria.^[^ ^32^ ^]^ We also demonstrated that PTPMT1 located in mitochondria in LUAD cells (Figure S8A, Supporting Information). Interestingly, we found that PTPMT1 interacted with SHMT2 and the interaction domain located in 304–404aa of SHMT2 (**Figure** **6**A,B, S8B, Supporting Information). Overexpressing PTPMT1 reduced the phosphorylation of SHMT2‐Ser90, and knocking down PTPMT1 increased this phosphorylation (Figure 6C–E). These results indicated that PTPMT1 dephosphorylates SHMT2 at Ser90. We subsequently investigated whether PTPMT1 regulates SHMT2 protein expression. Overexpressing PTPMT1 reduced the protein expression of SHMT2‐WT, while this effect was not observed for SHMT2‐S90A (Figure 6F). We detected the effects of PTPMT1 on SHMT2 protein stability. Overexpressing PTPMT1 significantly accelerated the protein degradation of SHMT2‐WT but did not affect the stability of SHMT2‐S90A (Figure 6G,H). We next examined whether PTPMT1 regulates the ubiquitination of SHMT2. Overexpressing PTPMT1 increased the ubiquitination of SHMT2‐WT, while the ubiquitination of SHMT2‐S90A was not affected (Figure 6I). We further demonstrated that overexpressing PTPMT1 induced K48‐linked ubiquitination of SHMT2 (Figure 6J,K, Figure S8C,D, Supporting Information), indicating that PTPMT1 regulated ubiquitination‐mediated protein degradation. As STUB1 mediates the ubiquitination and protein stability of SHMT2 phosphorylated at Ser90, we further explored the role of STUB1 in PTPMT1‐mediated ubiquitination. Knocking down STUB1 expression significantly reduced the ubiquitination of SHMT2 mediated by PTPMT1 (Figure S8E, Supporting Information). These results show that the PTPMT1‐mediated dephosphorylation and degradation of SHMT2 rely on the function of STUB1.

### 2.7 SHMT2‐Ser90 Phosphorylation Mediates Metabolic Reprogramming and m^6^A Modification of Global RNA

**Figure 7:**
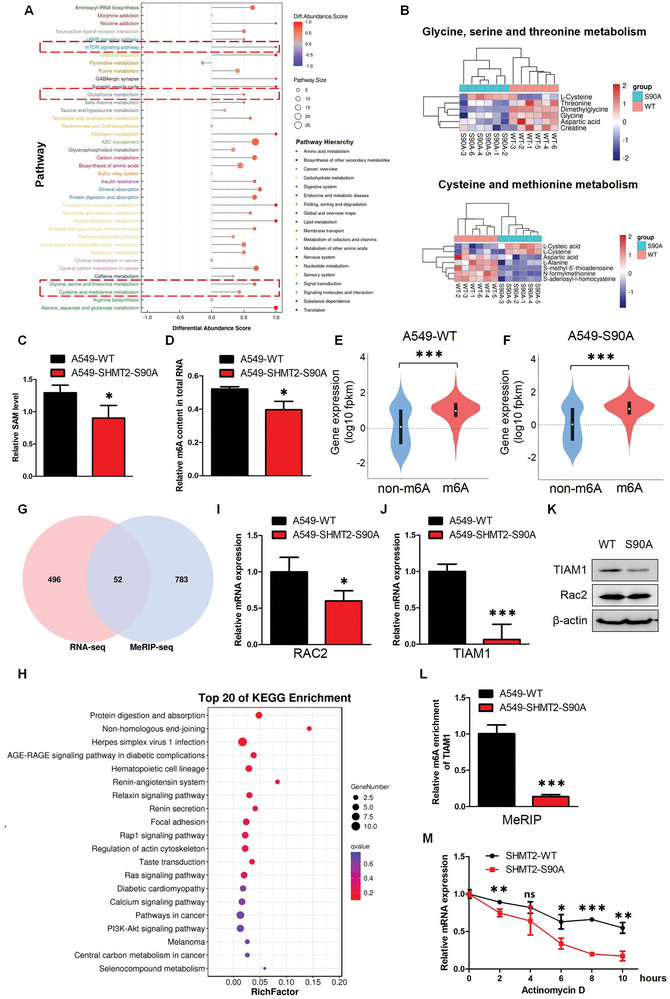
SHMT2‐Ser90 phosphorylation mediates metabolic reprogramming and m^6^A methylation of global RNA. A) KEGG pathway analysis of the metabolomics data. B) Cluster analysis of the metabolites involved in glycine, serine and threonine metabolism and cysteine and methionine metabolism. C) SAM levels in A549‐WT and A549‐SHMT2‐S90A cells were detected using a SAM detection kit. The data represent the average of three independent experiments (mean ± SD). *, *p* < 0.05. D) The m^6^A content in total RNA from A549‐WT and A549‐SHMT2‐S90A cells was detected using a m^6^A RNA methylation quantification kit. The data represent the average of three independent experiments (mean ± SD). *, *p* < 0.05. E,F) The correlations between m^6^A methylation and mRNA expression in A549‐WT (E) and A549‐SHMT2‐S90A (F) cells were analyzed using MeRIP‐Seq and RNA‐Seq data. Wilcoxon test was used for statistical evaluation. ***, *p* < 0.001. G) Differential genes identified from combined analysis of MeRIP‐Seq and RNA‐Seq data. H) KEGG pathway analysis of the DEGs identified from (G). I,J) The mRNA expression levels of RAC2 (I) and TIAM1 (J) were measured using Q‐PCR. The data represent the average of three independent experiments (mean ± SD). *, *p* < 0.05; ***, *p* < 0.001. K) Western blotting was used to detect the protein expression in A549‐WT and A549‐SHMT2‐S90A cells. L) The m^6^A levels in TIAMs from A549‐WT and A549‐SHMT2‐S90A cells were detected using a m^6^A RNA enrichment kit. The data represent the average of three independent experiments (mean ± SD). ***, *p* < 0.001. M) A549‐WT and A549‐SHMT2‐S90A cells were treated with actinomycin D for the indicated times, and Q‐PCR was performed to detect gene expression. ns, *p* > 0.05; *, *p* < 0.05; **, *p* < 0.01; ***, *p* < 0.001.

SHMT2 is a key enzyme involved in serine catabolism. To explore the effects of SHMT2‐Ser90 phosphorylation on tumor metabolism, we performed untargeted metabolomics to investigate the changes in global metabolites between A549‐WT and A549‐SHMT2‐S90A cells (Table S4, Supporting Information). We identified a total of 1164 metabolites in both the ESI‐positive and ESI‐negative modes using LC–MS/MS analysis (Figure S9A, Supporting Information). As shown in Figure S9B,C, Supporting Information, a series of metabolites with differential contents between A549‐WT and A549‐S90A cells were identified. KEGG pathway analysis was subsequently used to classify these differentially abundant metabolites. Most of the metabolic pathways, such as carbohydrate metabolism, amino acid metabolism and lipid metabolism, were downregulated in the A549‐SHMT2‐S90A cells. Specifically, in the amino acid metabolism pathway, glycine, which is the direct product of SHMT2, and N‐formylmethionine, which is closely related to the function of SHMT2 and account for mitochondrial initiator tRNA formylation,^[^ ^17^ ^]^ were significantly downregulated in A549‐SHMT2‐S90A cells (**Figure** **7**A,B). In the signal transduction pathway, the mTOR signaling pathway, which functions as the key regulator of metabolism, was downregulated in A549‐SHMT2‐S90A cells (Figure 7A). SHMT2 mediated one‐carbon metabolism is also involved in cellular redox homeostasis,^[^ ^3^ ^]^ and we found the changes in glutathione metabolism (Figure 7A). The precursors for glutathione synthesis like Glycine and γ‐Glu‐Cys were significantly decreased in A549‐SHMT2‐S90A cells (Table S4, Supporting Information). We then measured the ROS levels using DCFH‐DA and found that A549‐SHMT2‐S90A cells showed higher ROS levels than A549‐WT cells (Figure S9D, Supporting Information). The ratio of GSSG/GSH was also detected and Figure S9E, Supporting Information showed that GSSG/GSH ratio was increased in A549‐SHMT2‐S90A cells. These results demonstrated that the inhibition of SHMT2‐Ser90 phosphorylation inhibited the oncogenic metabolic pathway and simultaneously reduced the resistance of cancer cells to harmful stimuli. Methylation is an important modification that regulates the functions of DNA, RNA and proteins. S‐Adenosylmethionine (SAM) is the key substrate for methylation, and serine‐derived one‐carbon units are critical sources for SAM production.^[^ ^3^ ^]^ As shown in Figure 7B, the level of glycine, the direct product catabolized by SHMT2, and the metabolites related to SAM metabolism decreased in the A549‐SHMT2‐S90A cells. These findings indicated that inhibiting SHMT2‐Ser90 phosphorylation might affect SAM metabolism and methylation. The SAM level was measured, and Figure 7C shows that SAM was reduced in the A549‐SHMT2‐S90A cells. We next examined the methylation events in cells. DNA and protein methylation did not significantly differ between the A549‐WT and A549‐SHMT2‐S90A cells, while m^6^A modification decreased in the A549‐SHMT2‐S90A cells (Figure 7D, Figure S10A,B, Supporting Information). Then, methylated RNA immunoprecipitation sequencing (MeRIP‐Seq) and RNA‐Seq were performed to analyze the global changes in m^6^A modification and mRNA expression (Tables S5 and S6, Supporting Information). We found that the expressions of mRNAs with m^6^A were higher than that without m^6^A in both A549‐WT and A549‐SHMT2‐S90A cells (Figure 7E,F). Combined analysis of MeRIP‐Seq and RNA‐Seq was also conducted. A total of 835 genes with differential m^6^A peaks were identified via MeRIP‐Seq, and 548 DEGs were identified via RNA‐Seq. Among these genes, 52 exhibited changes in both m^6^A modification and mRNA expression (Figure 7G). These results were further analyzed using nine‐quadrant graph analysis, and the genes in quadrant 3 and quadrant 7, which were positively correlated with mRNA expression and m^6^A modification, were subjected to subsequent enrichment analysis via the KEGG database (Figure 7H and Figure S10C, Supporting Information). Among the significantly altered signaling pathways, the Ras signaling pathway was found. Because the Ras signaling pathway is also upstream of the mTOR pathway, which was identified via our metabolomics analysis, we next examined the expression of the genes in the Ras signaling pathway identified via the combined analysis. The mRNA expression levels of the identified genes Rac family small GTPase 2 (RAC2) and TIAM Rac1 associated GEF1 (TIAM1) were measured. Figure 7I,J shows that the mRNA expression levels of RAC2 and TIAM1 decreased in A549‐SHMT2‐S90A cells, but only TIAM1 protein expression was significantly reduced in A549‐SHMT2‐S90A cells (Figure 7K). We next examined the m^6^A enrichment of TIAM1 identified via MeRIP‐Seq. Figure 7L shows that m^6^A enrichment significantly decreased in A549‐SHMT2‐S90A cells. As m^6^A modification can affect RNA stability, we detected the stability of TIAM1 in A549‐WT and A549‐SHMT2‐S90A cells. Figure 7M shows that the TIAM1 mRNA was degraded much faster in the A549‐SHMT2‐S90A cells than in the A549‐WT cells. Finally, we examined the activation of the mTOR pathway in A549‐WT and A549‐SHMT2‐S90A cells. The results showed that the expression of p‐ERK, which is the key downstream protein of the Ras pathway, was downregulated. The expression of downstream targets of the mTOR pathway, including p‐S6K and p‐4EBP1, was also downregulated in A549‐SHMT2‐S90A cells, indicating that the mTOR pathway was inhibited (Figure S10D, Supporting Information). These results demonstrate that inhibiting the phosphorylation of SHMT2‐Ser90 prevents the m^6^A modification of key genes, leading to the inhibition of oncogenic metabolic pathways.

### 2.8 SHMT2‐Ser90 Phosphorylation Regulates LUAD Cell Tumorigenesis and is a Potential Diagnostic Marker for LUAD

**Figure 8:**
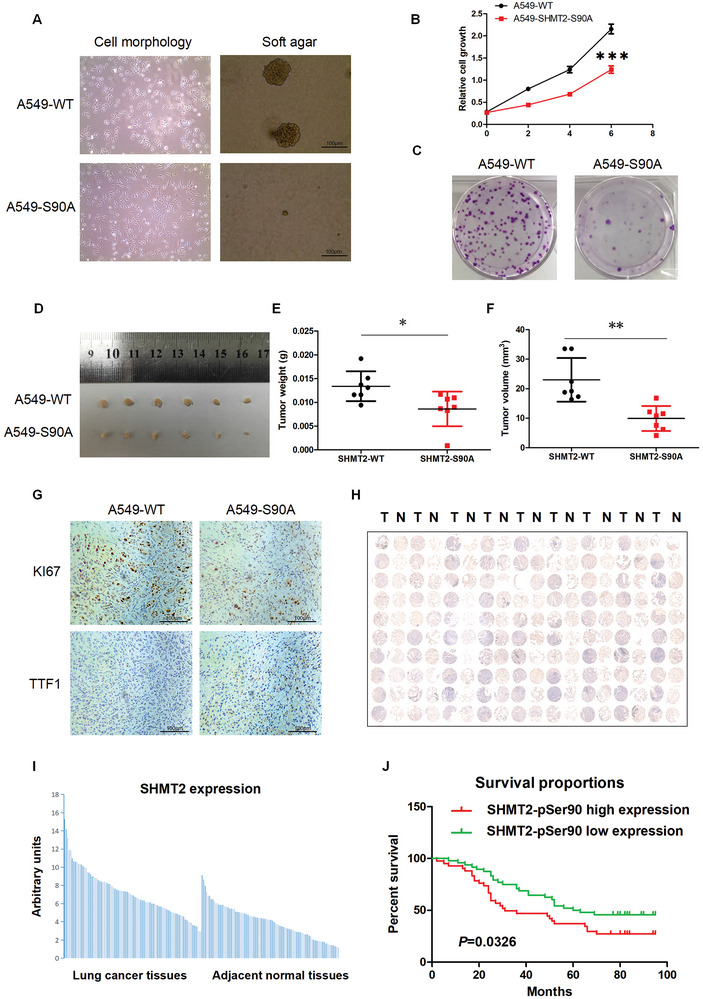
SHMT2‐Ser90 phosphorylation regulates LUAD cell tumorigenesis and is a potential diagnostic marker for LUAD. A) The morphology of A549‐WT and A549‐SHMT2‐S90A cells was observed using a microscope, and images were taken at 100× magnification (left panels). A soft agar assay was performed using A549‐WT and A549‐SHMT2‐S90A cells. On the 10th day, images were taken at 200× magnification (right panels). B) Cell proliferation was assessed in A549‐WT and A549‐SHMT2‐S90A cells. At the indicated times, cell proliferation was detected using crystal violet staining. ***, *p* < 0.001. C) A colony formation assay was performed using A549‐WT and A549‐SHMT2‐S90A cells. On Day 10, the cells were stained with crystal violet, and images were taken. D–F) A549‐WT or A549‐SHMT2‐S90A cells (1 × 10^7^) were subcutaneously injected into the flanks of nude mice. After 28 days, the tumors were dissected and photographed (D). The weight (E) and volume (F) of the xenograft tumors were measured. The P value was calculated by paired *t*‐test, **p < 0.01, *p < 0.05. G) Representative images of IHC staining of xenograft tumors for Ki67 (upper panels) and TTF1 (bottom panels) are shown. H) Microscopic evaluation of IHC staining of the LUAD tissue microarray using the SHMT2‐pSer90 antibody. N: adjacent normal tissue; T: tumor tissue. I) Quantification of the IHC staining shown in (H). J) Kaplan‒Meier survival curve of 90 patients in the LUAD tissue microarray. The patients were divided into two groups according to the average staining density of SHMT2‐pSer90 in the tumor tissues of the microarray (high expression: *n* = 42; low expression: *n* = 48; the log‐rank (Mantel‒Cox) test was used for the statistical analysis).

As SHMT2‐Ser90 phosphorylation regulates the metabolism of LUAD cells, we next investigated the function of this phosphorylation in tumor formation. Before we performed a xenograft assay, we discovered that the morphology of the A549‐SHMT2‐S90A cells was different from that of the A549‐WT cells, and a soft agar assay showed that the A549‐SHMT2‐S90A cells could not form colonies in soft agar, while the A549‐WT cells were not affected (**Figure** **8**A). Cell proliferation and colony formation assays showed that inhibiting the phosphorylation of SHMT2‐Ser90 reduced the proliferation rate and hindered colony formation (Figure 8B,C). Then, a xenograft assay was performed, and the results showed that the tumor volume and weight were decreased in the A549‐SHMT2‐S90A cell population (Figure 8D–F). We next detected the expression of genes indicative of the proliferation and differentiation states of lung adenocarcinoma cells by immunohistochemistry. Ki67 is widely used as a proliferation marker in pathological assessments, and NK2 homeobox 1, also known as thyroid factor‐1 (TTF‐1), was demonstrated to be frequently suppressed in high‐grade lung adenocarcinoma.^[^ ^33^, ^34^ ^]^ The immunohistochemistry results demonstrated that the expression of Ki67 was decreased in tumors derived from A549‐SHMT2‐S90A cells; however, TTF1 expression was higher in tumor‐derived A549‐SHMT2‐S90A cells than in A549‐WT cells (Figure 8G).

We also examined whether SHMT2‐Ser90 phosphorylation could be used as a diagnostic marker for LUAD. Another LUAD tissue microarray containing the survival information was used, and immunohistochemistry was performed using an anti‐SHMT2‐pS90 antibody. Figure 8H shows that most LUAD tissues had higher levels of phosphorylated SHMT2 than the adjacent normal tissues, and these findings were further validated by quantification of the staining (Figure 8I). These results were consistent with those in Figure S3C,D, Supporting Information. The statistical analysis of the quantified staining results from LUAD tissues divided the tumor samples into two groups according to the level of SHMT2‐pS90. Survival was subsequently correlated with the SHMT2‐pS90 level. As shown in Figure 8J, patients with low levels of phosphorylated SHMT2 exhibited better survival than patients with high levels (*p* = 0.0326). These results indicate that the phosphorylation of SHMT2 at Ser90 can be used as a potential diagnostic marker for LUAD.

## 3 Discussion

Metabolic reprogramming provides sufficient energy and metabolic intermediates for tumorigenesis and has become a promising area in the development of anticancer drugs.^[^ ^2^ ^]^ We discovered that the SHMT1/2 inhibitor SHIN1, which blocks serine catabolism, significantly hindered the proliferation of lung adenocarcinoma cells by using a screening compound library that directly inhibits metabolic enzymes. We clarified that the inhibitory effect of SHIN1 on lung adenocarcinoma cells was mediated mainly by SHMT2 but not by SHMT1. We also found that SHMT2 was upregulated in lung adenocarcinoma tissues and cells, while this change was not observed for SHMT1. These findings indicated that SHMT2 has more profound functions in lung adenocarcinoma cells. Previous studies have shown that SHMT1 knockdown inhibits the proliferation of lung cancer cells.^[^ ^19^ ^]^ We also found an inhibitory effect on cell proliferation by knocking out SHMT1 in lung adenocarcinoma cells. However, SHMT1 knockout had a much weaker inhibitory effect on proliferation than SHMT2 knockout. SHMT1 was also reported to be overexpressed in lung cancer cells. However, these studies either detected its expression only at the transcriptional level or examined its expression in a small number of cell lines.^[^ ^19^ ^]^ Our study examined SHMT1 protein expression in human bronchial epithelial cells and six lung adenocarcinoma cell lines and revealed that SHMT1 did not increase. Additionally, in the GEPIA platform, the transcription of SHMT1 was not increased in LUAD tissues. These results indicated that SHMT2 was the main isoform involved in mediating the proliferation of LUAD cells.

Posttranslational modifications are key mechanisms for regulating metabolic processes because of their rapid and reversible nature. Although SHMT2 is the key enzyme involved in serine catabolism, little is known about the functions of posttranslational modification in regulating SHMT2 in lung cancer. In fact, posttranslational modifications are important for the expression and activity of SHMT2 in some types of cancer. In colon cancer, acetylation of Lys95 in SHMT2 disrupted its tetramer structure and inhibited its enzymatic activity; this acetylation also promoted SHMT2 degradation through the ubiquitin‐lysosome pathway. SIRT3‐mediated deacetylation of SHMT2 promoted colorectal carcinogenesis.^[^ ^14^ ^]^ Another study showed that SIRT5‐mediated desuccinylation of SHMT2 increased its enzymatic activity and drove serine catabolism in colon and osteosarcoma cancer cells.^[^ ^27^ ^]^ These two modifications exerted functional inhibitory effects. Until recently, the functional activation modification of SHMT2 was unknown. In this study, we found that SHMT2 can be phosphorylated at serine, threonine and tyrosine residues, and only serine phosphorylation was increased in LUAD cells compared with that in human bronchial epithelial cells. We elucidated that Ser90 of SHMT2 was the key phosphorylation site regulating its protein stability. However, the enzymatic activity was not affected by this phosphorylation. We further demonstrated that MAPK1 was the kinase responsible for the phosphorylation of SHMT2 at Ser90. The MAPK/ERK pathway, which is critical for cellular responses to extracellular stimuli, has been demonstrated to be a key regulator of metabolism during tumorigenesis.^[^ ^35^ ^]^ Constitutive activation of the MAPK/ERK pathway frequently occurs in lung adenocarcinoma due to mutations in genes such as EGFR or KRAS.^[^ ^36^, ^37^ ^]^ However, whether the MAPK/ERK pathway regulates serine metabolism has not been determined. We demonstrated for the first time that MAPK1 regulates serine catabolism through phosphorylating SHMT2 in lung adenocarcinoma. Notably, in our in vitro kinase assay, MAPK1 also phosphorylated the SHMT2‐S90A mutant. These findings indicate that MAPK1 can also phosphorylate other serine residues in addition to Ser90 to regulate the function of SHMT2. Another interesting finding in our study was that STUB1 overexpression only induced a slight decrease in SHMT2‐WT protein levels. We thought that although SHMT2‐WT underwent dynamic processes of phosphorylation and dephosphorylation in cancer cells, the phosphorylation state may be dominant because of the constitutive activation of MAPK1 pathway in cancer cells and the dephosphorylated SHMT2 could be rapidly rephosphorylated on Ser90. Thus, the endogenous STUB1 was sufficient to degrade the non‐phosphorylated SHMT2 and overexpressing STUB1 was unable to further accelerate the degradation rate of SHMT2‐WT. Thus, our study demonstrated that the MAPK/ERK pathway activates serine catabolism through phosphorylating and stabilizing SHMT2, contributing to the biosynthesis of macromolecules and functional modification (methylation) for cancer progression.

PTPMT1 (protein tyrosine phosphatase mitochondrial 1) was originally cloned on the basis of the similarity of its catalytic site with that of PTEN.^[^ ^38^ ^]^ The mitochondrial localization of PTPMT1 indicated that it may regulate metabolic processes. PTPMT1 was initially demonstrated to have strong activity against the phosphoinositide PI5P in vitro.^[^ ^32^ ^]^ However, as a protein phosphatase, little is known about its protein substrates, especially metabolic enzymes. It was reported that succinate dehydrogenase (SDH) is a PTPMT1 substrate. PTPMT1 can dephosphorylate SDH at serine and tyrosine residues.^[^ ^39^ ^]^ Here, we discovered that SHMT2 is a new substrate of PTPMT1. PTPMT1 can dephosphorylate SHMT2 at Ser90, leading to the ubiquitination and degradation of SHMT2 by STUB1. This finding expands the known functions of PTPMT1 in regulating mitochondrial metabolism, and additional studies are needed to further elucidate the roles of PTPMT1 in cancer metabolism.

In addition to providing building blocks, another important function of serine catabolism is providing one‐carbon units for methylation in cancer cells. In this study, we investigated the effects of SHMT2‐Ser90 phosphorylation on intracellular methylation events, including DNA methylation, protein methylation and RNA m^6^A methylation. To our surprise, we found that although the total SAM level decreased when SHMT2‐Ser90 phosphorylation was inhibited, global DNA and protein methylation were not significantly affected. Only global m^6^A methylation was significantly decreased. Combined with our RNA‐Seq analysis, these findings revealed that m^6^A methylation was associated with RNA expression. We further demonstrated that increased m^6^A methylation enhances RNA stability and the expression of key genes in oncogenic pathways, leading to cancer progression. An interesting question that needs to be further answered is why SHMT2‐Ser90 phosphorylation affects only the m^6^A methylation of total RNA and how this selectivity is achieved in lung adenocarcinoma. Another limitation of this study is that m^6^A methylation has a wide range of functions in addition to RNA degradation, including RNA processing, RNA export and translation, and is also involved in chromatin and transcriptional regulation.^[^ ^40^ ^]^ However, the influence of SHMT2 phosphorylation on these biological processes needs to be further clarified. In addition, unchanged methylation levels of total protein and DNA do not represent changes in a certain gene. The influence of SHMT2 phosphorylation on the protein or DNA methylation of certain oncogenic genes is an intriguing topic.

**Figure 9:**
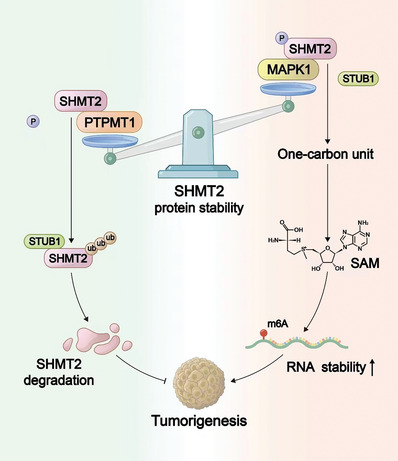
The working model of this study.

In conclusion, our study clarified that MAPK1 and PTPMT1 cooperatively regulate the phosphorylation and protein stability of SHMT2. MAPK1 phosphorylates SHMT2 at Ser90, and this phosphorylation enhances its protein stability, leading to increased SAM production and RNA m^6^A modification. This modification enhances the RNA stability of cancer‐promoting genes, thus promoting tumorigenesis. In contrast, PTPMT1 dephosphorylates SHMT2 at Ser90, followed by STUB1‐mediated ubiquitination and proteasomal degradation of SHMT2, resulting in the inhibition of tumorigenesis (**Figure** **9**).

## 4 Experimental Section

### Reagents and Plasmids

The screening library for the inhibition of metabolic enzymes was obtained from MCE (HY‐L0001). The kinase inhibitors BDP5290 (HY‐12437), LY3214996 (HY‐101494), PIM447 (HY‐19322), seliciclib (HY‐30237), tomivosertib (HY‐100022), and DMAT (HY‐15535) were purchased from MCE. H2DCFDA (DCFH‐DA) was purchased from MCE (HY‐D0940). GSH and GSSG assay kit was ordered from Beyotime (S0053). Cycloheximide (CHX) and chloroquine (CQ) were purchased from Sigma (C7698, C6628). MG132 was obtained from Selleck (S2619). The STUB1 and PTPMT1 siRNAs were purchased from Thermo Fisher Scientific (1 299 001). The UBR5 siRNAs were purchased from OriGene (SR309739). The MAPK1 siRNAs were purchased from TSINGKE. We constructed the pcDNA3.1‐HA‐SHMT2 and pCMV‐HA‐STUB1 plasmids ourselves. The pCMV6‐Flag‐STUB1 and pCMV6‐Flag‐STUB1‐H260Q plasmids were constructed by MiaolingBio. The SHMT2 phosphorylation mutants (S76A, S90A, Y106F, S266A, and T420A) were constructed by TSINGKE. The pcDNA3.1‐Flag‐SHMT2‐S90A lysine mutants (K95R, K103R, K181R, K200R, K262R, K269R, K280R, K297R, K356R, K389R, K409R, K464R, K469R, and K474R) and pcDNA3.1‐Flag‐SHMT2 truncations (wild‐type, 1–404aa, 1–304aa, 1–204aa) were constructed by SynbioB. The pCMV3‐Myc‐MAPK1 (HG10030‐CM) and Myc‐MTHFD2 (HG16324‐CM) plasmids were obtained from SinoBiological. The pCMV6‐Flag‐PTPMT1 was obtained from OriGene (RC215376). The UB‐K48R and UB‐K63R mutants were kindly provided by Dr. Caifeng Xie at Nanchang University. For the antibodies, SHMT2 (11099‐1‐AP), β‐actin (66009‐1‐Ig), SHMT1 (30192‐1‐AP), Myc‐tag (60003‐2‐Ig), HUWE1 (19430‐1‐AP), STUB1 (55430‐1‐AP), UBR5 (66937‐1‐Ig), MAPK1 (51068‐1‐AP), ubiquitin (10201‐2‐AP), PTPMT1 (11493‐1‐AP), RAC2 (10735‐1‐AP), TIAM1 (27694‐1‐AP), phospho‐ERK1/2 (Thr202/Tyr204) (28733‐1‐AP), S6K (14485‐1‐AP), phospho‐S6K (Thr389) (28735‐1‐AP) and 4EBP1 (60246‐1‐Ig) antibodies were purchased from Proteintech. The mouse anti‐HA tag monoclonal antibody was obtained from Thermo Fisher Scientific (26 183). Mouse anti‐Flag tag monoclonal antibody and phosphoserine antibody were obtained from Sigma‐Aldrich (F1804, P5747). The phospho‐S6K (Ser65) (9451) antibody, K48‐linkage‐specific polyubiquitin antibody (8081) and K63‐linkage‐specific polyubiquitin antibody (5621) were purchased from Cell Signaling Technology. Phosphothreonine (PTM‐705RM), phospho‐tyrosine (PTM‐702RM), Symmetric Di‐Methyl Arginine (PTM‐617RM), Asymmetric Di‐Methyl Arginine (PTM‐605RM, Mono/Di‐Methyllysine (PTM‐602), and Tri‐Methyllysine (PTM‐601) antibodies were obtained from PTMBio. The anti‐MKI67/Ki67 and anti‐NKX2‐1/TTF1 antibodies were purchased from Abcam (ab15580, ab76013). Phospho‐SHMT2 (Ser90) was generated by Shanghai Genomics, Inc. The protein G agarose beads were purchased from Roche (11 243 233 001).

### Cell Culture

Human bronchial epithelial cells (BEAS‐2B) were purchased from the National Collection of Authenticated Cell Cultures and cultured in RPMI 1640 (Gibco) supplemented with 10% FBS (Excell). Human lung adenocarcinoma cells (H1299, A549, H1975, H358, H23, HCC827 and PC9) purchased from the National Collection of Authenticated Cell Cultures were cultured in RPMI 1640 (Gibco) supplemented with 10% FBS (Excell). HEK‐293T cells were purchased from the National Collection of Authenticated Cell Cultures and cultured in DMEM (Dulbecco's Modified Eagle Medium, Gibco) supplemented with 10% FBS (Excell). A549 cells with the endogenous SHMT2‐S90A mutation were generated via the CRISPR/Cas9 technique by Cyagen Biosciences. The cells were cultured in RPMI 1640 (Gibco) supplemented with 10% FBS (Excell). All the cells were cultured under 5% CO_2_ at 37 °C.

### Cell Growth Assay

For compound library screening, cells were seeded in 96‐well plates at 5000 cells per well in 100 µL of medium supplemented with 10% FBS. On the following day, the medium was changed to RPMI 1640+10% FBS+inhibitors. After 48 h, CCK8 was added, and the cells were incubated for 1 h in 5% CO_2_ at 37 °C. Then, the absorbance at 490 nm was detected. The inhibition rates were used to plot the images.

For the cell proliferation assay, cells were seeded in 24‐well plates at 3000 cells per well in 500 µL of medium supplemented with 10% FBS. On the following day, media supplemented with different concentrations of SHIN1 were added. The medium was changed every 2 days. At the indicated times, the cells were fixed in 3.7% formaldehyde and stained with 0.1% crystal violet. Dye was extracted with 10% acetic acid, and the relative proliferation was determined by the absorbance at 595 nm.

To measure growth inhibition rates, cells were seeded in 96‐well plates at 5000 cells per well in 100 µL of medium supplemented with 10% FBS. On the following day, different concentrations (0, 0.625, 1.25, 2.5, 5, and 10 µm) of SHIN1 were added. 44 h later, 100 µL of medium supplemented with 10 µL of MTT was added to the wells, which were then incubated for 4 h in 5% CO_2_ at 37 °C. Then, the medium was discarded, and 100 µL of DMSO was added to the wells. After mixing on an endover shaker for 15 minutes, the absorbance at 490 nm was detected. The log values of the drug concentrations were used as the X axis, and the corresponding inhibition rates of cell proliferation were used as the Y axis. The images were created using GraphPad software.

For the colony formation assay, cells were seeded in 6‐well plates at a density of 500 cells per well in 1 mL of medium supplemented with 10% FBS. The medium was changed every 2 days. After 10 days, the cells were fixed in 4% formaldehyde for 30 min and stained with 0.1% crystal violet. The images were obtained using a digital camera (Canon, EOS70D).

For the soft agar assay, 10^4^ cells were suspended in RPMI 1640 supplemented with 10% FBS and 0.3% agarose, after which the cells were plated on the top of a solidified layer of RPMI 1640 supplemented with 0.5% agarose and 10% FBS. Fresh medium supplemented with 10% FBS and 0.5% agarose was added to the cells every week. 2 weeks later, images were taken using an Olympus IX71 microscope.

### Patient‐Derived Lung Adenocarcinoma Organoids

The tumor tissues from lung adenocarcinoma patients were washed with cold HBSS. The tissues were cut into small pieces, digested with Tumor Tissue Digestion Solution (BIOGENOUS BIOTECHNOLOGY, INC., Cat No. K601003) and centrifuged to collect the tumor fraction. These tumor fractions were embedded in Matrigel (BD Biosciences, 356 231) and seeded on 24‐well plates. After Matrigel polymerization, culture medium (BIOGENOUS BIOTECHNOLOGY, INC., Cat No. K2138‐LA) was added, and the medium was refreshed every 2 days. For SHIN1 treatment, culture medium supplemented with 20 µm SHIN1 was added, and the medium was refreshed every 2 days. On Day 7, images were taken using an Olympus IX71 microscope. All the experiments involving human tissue samples were approved and supervised by the Ethics Committee of the First Affiliated Hospital of Nanchang University, with No. (2023)CDYFYYLK(04‐003).

### RNA Interference and Gene Overexpression

For RNA interference, cells were transiently transfected with the siRNAs using SuperFectin siRNA Transfection Reagent (Pufei, 2103–100) following the manufacturer's instructions. After 48 h, the transfection efficiency was checked by western blot analysis using the relevant antibodies. For gene overexpression, cells were transiently transfected with the indicated plasmids using the SuperFectin DNA Transfection Reagent Kit (Pufei, 2102–100) following the manufacturer's instructions. 48 h later, the transfection efficiency was checked by western blotting using the indicated antibodies.

### CRISPR‒Cas9‐Mediated Gene Knockout

The sgRNA sequences targeting SHMT1/2 were designed by the CRISPR designer at https://chopchop.cbu.uib.no/. The sgRNA sequences were subsequently cloned and inserted into the lentiCRISPR‐v2‐Flag vector. HEK‐293T cells (5 × 10^5^) were seeded in a 6‐well plate. After 24 h, the cells were transfected with the packaging construct psPAX2 (2 µg), the lentiviral vector pMD2. G (1.5 µg), and lentiCRISPR‐v2‐Flag‐SHMT1/2 (1 µg) using SuperFectin DNA Transfection Reagent. At the indicated times, the supernatants containing the virus particles were collected, filtered through a 0.45‐µm filter, and subsequently used to infect the A549 cells. 24 h later, the cells were selected with 10 µg mL^−1^ puromycin (Solarbio, P8230). The surviving clones were transferred individually into 96‐well plates. The knockout efficiency was determined by western blotting. The CRISPR‒Cas9 plasmid targeting a tomato gene was used as a negative control.

### Quantitative RT‒PCR

Total RNA was extracted using RNAiso Plus reagent (Takara, 9109), and 1 µg of total RNA was reverse transcribed using a PrimeScript RT reagent kit with gDNA eraser (Takara) according to the manufacturer's instructions. Quantitative RT‒PCR was performed with SYBR Green dye. The relative amount of cDNA was calculated by the comparative Ct method using GAPDH as a control. The probe sequences were: SHMT2:5′‐CCCTTCTGCAACCTCACGAC‐3′(sense); 5′‐TGAGCTTATAGGGCATAGACTCG‐3′(antisense); RAC2:5′‐CAACGCCTTTCCCGGAGAG‐3′(sense); 5′‐TCCGTCTGTGGATAGGAGAGC‐3′(antisense); TIAM1:5′‐GATCCACAGGAACTCCGAAGT‐3′(sense); 5′‐GCTCCCGAAGTCTTCTAGGGT‐3′(antisense). PCRs were performed in triplicate.

### Immunoprecipitation and Western Blotting

For the immunoprecipitation assay, cells were lysed in NP‐40 lysis buffer (150 mm NaCl, 20 mm β‐glycerol phosphate, 1 mm Na orthovanadate, 20 mm NaF, 0.5% Nonidet P‐40, 20 mm HEPES, pH 7.4) containing a protease inhibitor cocktail (Sigma, P2714), phenylmethylsulfonyl fluoride (DINGGUO, WB0180) and phosphatase inhibitor cocktail (Beyotime, P1082) for 30 min at 4 °C. Then, the cell lysates were centrifuged at 10 000× g for 20 min at 4 °C. The indicated antibodies and protein G agarose beads were added to the supernatants, which were subsequently incubated overnight at 4 °C. The mixtures were subsequently washed with lysis buffer three times, suspended in 2× loading buffer and boiled for 10 min.

For western blotting, cells were lysed in NP‐40 lysis buffer containing protease inhibitors, phenylmethylsulfonyl fluoride and phosphatase inhibitors, and the protein concentrations were measured with a BCA Protein Assay Kit (Pierce Biotechnology, 23 225). Total proteins were subjected to 10% or 12% SDS‒PAGE and transferred to PVDF membranes (Millipore, IPVH00010). The membranes were blocked with 5% skim milk (BD, 232 100) or BSA (Genview, FA‐016) for 1 h at room temperature and incubated with the indicated antibodies overnight at 4 °C. The membranes were washed three times at room temperature with 1× TBST (20 mm Tris‐base [Solarbio, T8060], 150 mm NaCl [SCR, 10 019 318], 0.05% Tween‐20 [Solarbio, T8220], pH 7.4) for 10 min each, followed by incubation for 1 h at room temperature with horseradish peroxidase‐conjugated anti‐mouse secondary antibodies (Thermo Fisher Scientific, 31 430) or anti‐rabbit secondary antibodies (Thermo Fisher Scientific, 31 460). Protein bands were visualized after incubation with a Pro‐Light chemiluminescence detection kit (TIANGEN, PA112‐01) using the digital gel image analysis system TANON 5500, and the band intensities were quantified with Tanon GIS software.

### In Vitro Kinase Assay

HA‐SHMT2 immunoprecipitated from BEAS‐2B cells and MYC‐MAPK1 immunoprecipitated from A549 cells were used to perform an in vitro kinase assay. The immunoprecipitates were washed once with kinase reaction buffer containing 50 mm Tris‐HCl (pH 7.5), 2 mm EGTA, 10 mm MgCl_2_, 0.1 mm DTT, and 0.1% Triton X‐100. Then, exogenous HA‐SHMT2 and MYC‐MAPK1 were added to kinase reaction buffer containing 100 µm ATP. The reaction proceeded for 30 min at 30 °C with gentle rocking and was terminated by adding 1× SDS sample buffer and heating at 95 °C for 5 min. Western blotting was used to detect phosphorylation via the use of SHMT2‐pS90 and p‐Ser antibodies.

### SHMT2 Activity Assay

The detailed procedures for the SHMT2 activity assay were carried out as previously described.^[^ ^27^ ^]^ Briefly, exogenous HA‐SHMT2 and Myc‐MTHFD2 were transfected into A549 cells, which were immunoprecipitated using anti‐HA or anti‐Myc antibodies. The immunoprecipitates were added to reaction buffer containing 50 mm Tris‐HCl (pH 8.0), 100 mm NaCl, 5 mm NADP^+^, 2 mm NAD^+^, 2 mm THF, 0.5 mm serine, 0.5 mm PLP, 5 mm MgCl_2_, 1 mg mL^−1^ BSA and 10% glycerol. The reactions proceeded for 30 min at 37 °C, after which the absorbance at 350 nm was measured.

### Measurements of DNA Methylation

The DNA methylation levels were measured using a MethylFlash Global DNA Methylation (5‐mC) ELISA Easy Kit (colorimetric) purchased from EpigenTec (p‐1030). One hundred nanograms of genomic DNA was used for each reaction. The assays were performed following the protocol described in the instruction manuals.

### Measurements of S‐Adenosyl Methionine

The SAM levels were measured using a SAM ELISA kit purchased from ELK Biotechnology (ELK8108). The assays were performed following the protocol described in the instruction manuals.

### Measurements of m^6^A Content in Total RNA

The m^6^A content was detected using an EpiQuik m^6^A RNA Methylation Quantification Kit (colorimetric) purchased from EpigenTec (p‐9005). Total RNA (200 ng) was used for each reaction. The assays were performed following the protocol described in the instruction manuals.

### Measurements of m^6^A Enrichment in TIAM1 RNA

m^6^A enrichment in TIAM1 RNA was detected using an EpiQuik CUT&RUN m^6^A RNA Enrichment (MeRIP) kit purchased from EpigenTec (p‐9018). Total RNA (10 µg) was used for each reaction. The assays were performed following the protocol described in the instruction manuals.

The m^6^A modification sequence of TIAM1 was identified via MeRIP‐seq (Table S5, Supporting Information). The sequences of primers used for Q‐PCR quantification were 5′‐AAGAGCGCGACCTAA‐3′ (sense) and 5′‐CGCAAGCGAGGAAGT‐3′ (antisense).

### In Vivo Xenograft Assay

Cell suspensions (1 × 10^7^ cells) in a total volume of 100 µL were injected subcutaneously into the flanks of ≈3–4‐week‐old male BALB/C nude mice (GemPharmatech). 4 weeks after the injection, the mice were sacrificed, the tumors were dissected, and the weights and volumes were measured. Tumor volume was calculated with the following formula: volume (mm3) = π/6 × (large diameter) × (smaller diameter)^2^. All mice lived in the SPF animal facility of the Institute of Translational Medicine at Nanchang University, and the experimental procedures were approved by the Institutional Animal Use and Care Committee of Nanchang University (NCULAE‐20221130009).

### Immunohistochemistry

A lung adenocarcinoma tissue microarray was separately purchased from the National Engineering Center for BioChips and Servicebio. A more complete description of the human specimens is included in Tables S7 and S8, Supporting Information. The expression of SHMT2 and phosphorylated SHMT2 in the tissue was evaluated by immunohistochemical staining with SHMT2‐ and SHMT2‐pS90‐specific antibodies. The tissue microarray slide was deparaffinized, rehydrated, and subjected to an epitope retrieval step. Subsequently, 6% hydrogen peroxide was used to block endogenous peroxidase activity. The slides were washed in PBS (DINGGUO, BF‐0011) three times and then incubated with SHMT2‐pS90 or SHMT2 antibodies at 4 °C overnight. After three washes in PBS, the slides were incubated with horseradish peroxidase‐conjugated secondary antibody (Servicebio, GB23303) for 1 h. The stain was developed with either chromogen or hematoxylin solution. The images of the tissue microarray were analyzed by ImageScope software. Immunohistochemical staining of tumors derived from A549‐WT and A549‐SHMT2‐S90A cells was performed as described above. Images were taken using an Olympus IX71 microscope.

### Mitochondria Isolation

Mitochondria were isolated using Qproteome Mitochondria Isolation kit (QIAGEN, 37 612) following the manufacturer's instructions.

### Untargeted Metabolomics

Untargeted metabolomics was performed and analyzed by Applied Protein Technology.

### Identification of Phosphorylation Sites

The HA‐SHMT2 plasmid was transfected into A549 cells, which were subsequently immunoprecipitated using anti‐HA antibodies. The identification of phosphorylation sites on SHMT2 was performed, and the proteins were analyzed by QLBio using mass spectrometry.

### Identification of the Binding Proteins

The HA‐SHMT2 and HA‐SHMT2‐S90A plasmids were transfected into A549 cells, which were subsequently immunoprecipitated using anti‐HA antibodies. The identification of the proteins that bind to SHMT2 was performed in Bioprofile via mass spectrometry.

### MeRIP‐seq and RNA‐seq

MeRIP‐seq and RNA‐seq were conducted and analyzed by Gene Denovo.

### Statistical Analysis

GraphPad Prism 8 software was used for statistical analysis. Unless otherwise stated, the data represent the average of at least three independent experiments (mean ± SD). Student's *t*‐test or one‐way ANOVA was used for statistical evaluation, and *p* values ≤ 0.05 were considered to indicate statistical significance.

## Conflict of Interest

The authors declare no conflict of interest.

## Author Contributions

T.H. designed the study. T.H. and J.W. analyzed and interpreted the data. T.H., Y.W., M.C., Q.H., X.W., M.H., Y.L., W.X., J.X., L.W., R.L., Y.Y., and K.W. performed the experiments. T.H. and J.W. wrote the manuscript. This manuscript was approved by all authors.

## Supporting information

Supporting Information

Supplemental Table 1

Supplemental Table 2

Supplemental Table 3

Supplemental Table 4

Supplemental Table 5

Supplemental Table 6

Supplemental Table 7

Supplemental Table 8

## Data Availability

The data that support the findings of this study are available from the corresponding author upon reasonable request.
