# Supplementary material for: Phosphorylated SHMT2 Regulates Oncogenesis Through m6A Modification in Lung Adenocarcinoma
Source: Adv Sci (Weinh). 2024 Mar 9;11(18):2307834. doi: 10.1002/advs.202307834 (PMC11095143; doi:10.1002/advs.202307834)
Supplement: Supplementary file 1 — Supporting Information [file ADVS-11-2307834-s004.pdf]

ADVANCED SCIENCE
Open Access

Supporting Information

for *Adv. Sci.*, DOI 10.1002/advs.202307834

Phosphorylated SHMT2 Regulates Oncogenesis Through m^6A Modification in Lung Adenocarcinoma

Tianyu Han, Yanan Wang, Minzhang Cheng, Qifan Hu, Xiaorui Wan, Menglin Huang, Yuhan Liu, Wenze Xun, Jin Xu, Lei Wang, Ruiguang Luo, Yi Yuan, Keru Wang and Jianbin Wang**

Supplementary information

Figures and Figure legends

Fig.S1

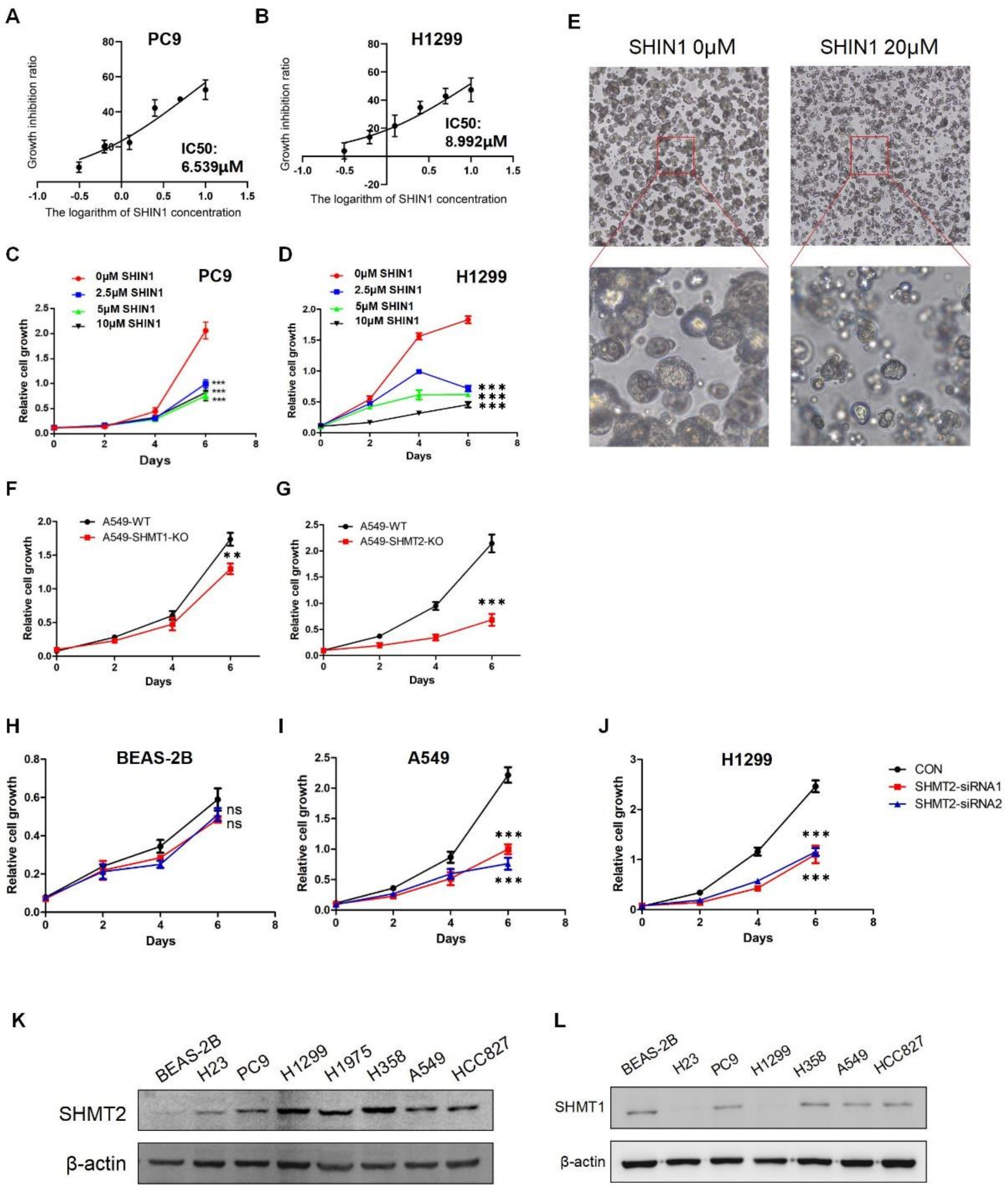

Figure S1. SHIN1 inhibits the proliferation of LUAD cells. (**A and B**) Different concentrations of SHIN1 were used to treat PC9 (A) and H1299 cells (B) for 48 hours. The growth inhibition rates were detected using

MTT assay. Data represent the average of three independent experiments (mean±SD). (**C and D**) Different concentrations of SHIN1 were used to treat PC9 (C) and H1299 cells (D) for the indicated times. Cell proliferation was detected using crystal violet staining. Data represent the average of three independent experiments (mean±SD). ***, P<0.001. (**E**) patient-derived lung adenocarcinoma organoids were treated with 20μM SHIN for 7 days. The photographs were taken under 40× and 100× magnification. (**F and G**) The cell proliferation rates of A549-SHMT1-KO (F) and A549-SHMT2-KO (G) were detected using crystal violet staining. Data represent the average of three independent experiments (mean±SD). **, P<0.01; ***, P<0.001. (**H-J**) The cell proliferation rates of BEAS-2B (H), A549 (I) and H1299 (J) cells were detected using crystal violet staining. Data represent the average of three independent experiments (mean±SD). ns, P>0.05; ***, P<0.001. (**K**) The protein expressions of SHMT2 in human bronchial epithelial cells (BEAS-2B) and LUAD cells (H23, PC9, H1299, H1975, H358, A549, HCC827) were examined using western blot. (**L**) The protein expressions of SHMT1 in human bronchial epithelial cells (BEAS-2B) and LUAD cells (H23, PC9, H1299, H358, A549, HCC827) were examined using western blot.

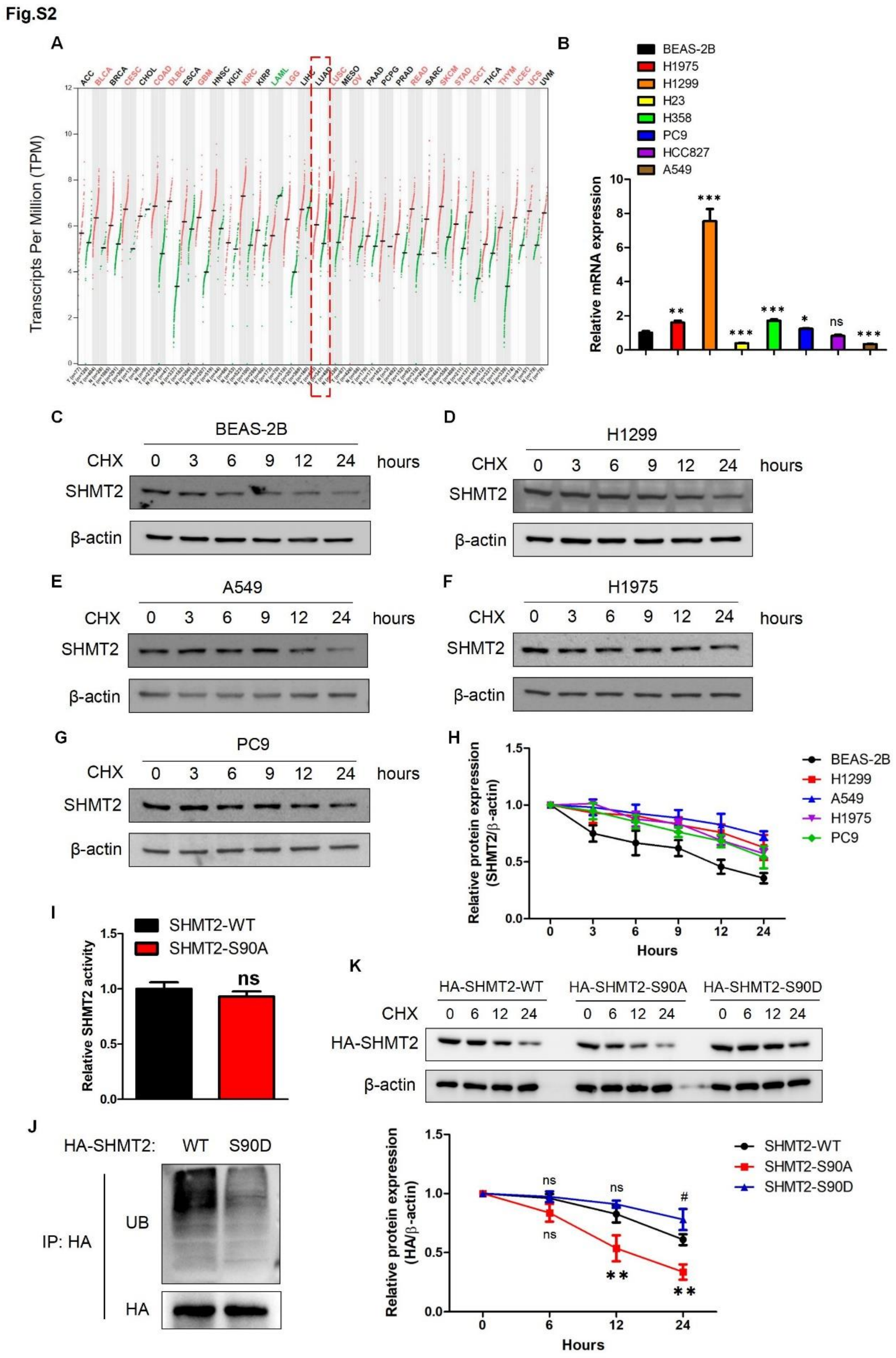

Figure S2. The expressions of SHMT2 in LUAD cells are mainly regulated by protein stability. (**A**) The mRNA levels of SHMT2 in

cancer tissues and normal lung tissues were analyzed using GEPIA platform. The cancer types labled in red indicated up-regulation of mRNA levels in cancer tissues, the cancer types labled in green indicated down-regulation of mRNA levels in cancer tissues and the cancer types without color annotation indicated no significant changes in mRNA expression. The red dashed line indicates the mRNA expression of SHMT2 in lung adenocarcinoma. (**B**) The mRNA levels of SHMT2 in human bronchial epithelial cells (BEAS-2B) and LUAD cells (H23, PC9, H1299, H1975, H358, A549, HCC827) were examined using Q-PCR. Data represent the average of three independent experiments (mean±SD). ns, P>0.05; *, P<0.05; **, P<0.01; ***, P<0.001. (**C-G**) CHX was used to treated human bronchial epithelial cells (BEAS-2B) and LUAD cells (H1299, A549, H1975, PC9) for indicated times and western blot was used to detect the expression of SHMT2. (H) Relative SHMT2 expression over β-actin was quantified. The data represent the average of three independent experiments (mean±SD). (BEAS-2B vs H1299: 3h, P<0.05; 6h, P<0.01; 9h, P<0.01; 12h, P<0.001; 24h, P<0.01. BEAS-2B vs A549: 3h, P<0.01; 6h, P<0.01; 9h, P<0.001; 12h, P<0.001; 24h, P<0.001. BEAS-2B vs H1975: 3h, P<0.05; 6h, P<0.05; 9h, P<0.01; 12h, P<0.01; 24h, P<0.05. BEAS-2B vs PC9: 3h, P<0.05; 6h, P<0.05; 9h, P<0.05; 12h, P<0.01; 24h, P<0.05). (I) HA-SHMT2-WT and HA-SHMT2-S90A were transfected into A549 cells, which were subsequently immunoprecipitated

using an anti-HA antibody. The SHMT2 activity was detected. The data represent the average of three independent experiments (mean±SD). ns, P>0.05. (J) HA-SHMT2-WT and HA-SHMT2-S90D were separately transfected into A549 cells. Forty-eight hours later, immunoprecipitation was performed, and ubiquitination was detected. (K) Wild-type SHMT2 and SHMT2 mutants (S90A, S90D) were transfected into A549 cells. CHX was used to treat the cells for the indicated times, and protein degradation was detected via western blotting (Top panel). Relative HA-SHMT2 expression over β-actin was quantified. The data represent the average of three independent experiments (mean±SD). #, S90D vs WT; *, S90A vs WT. ns, P>0.05; **, P<0.01; #, P<0.05 (Bottom panels).

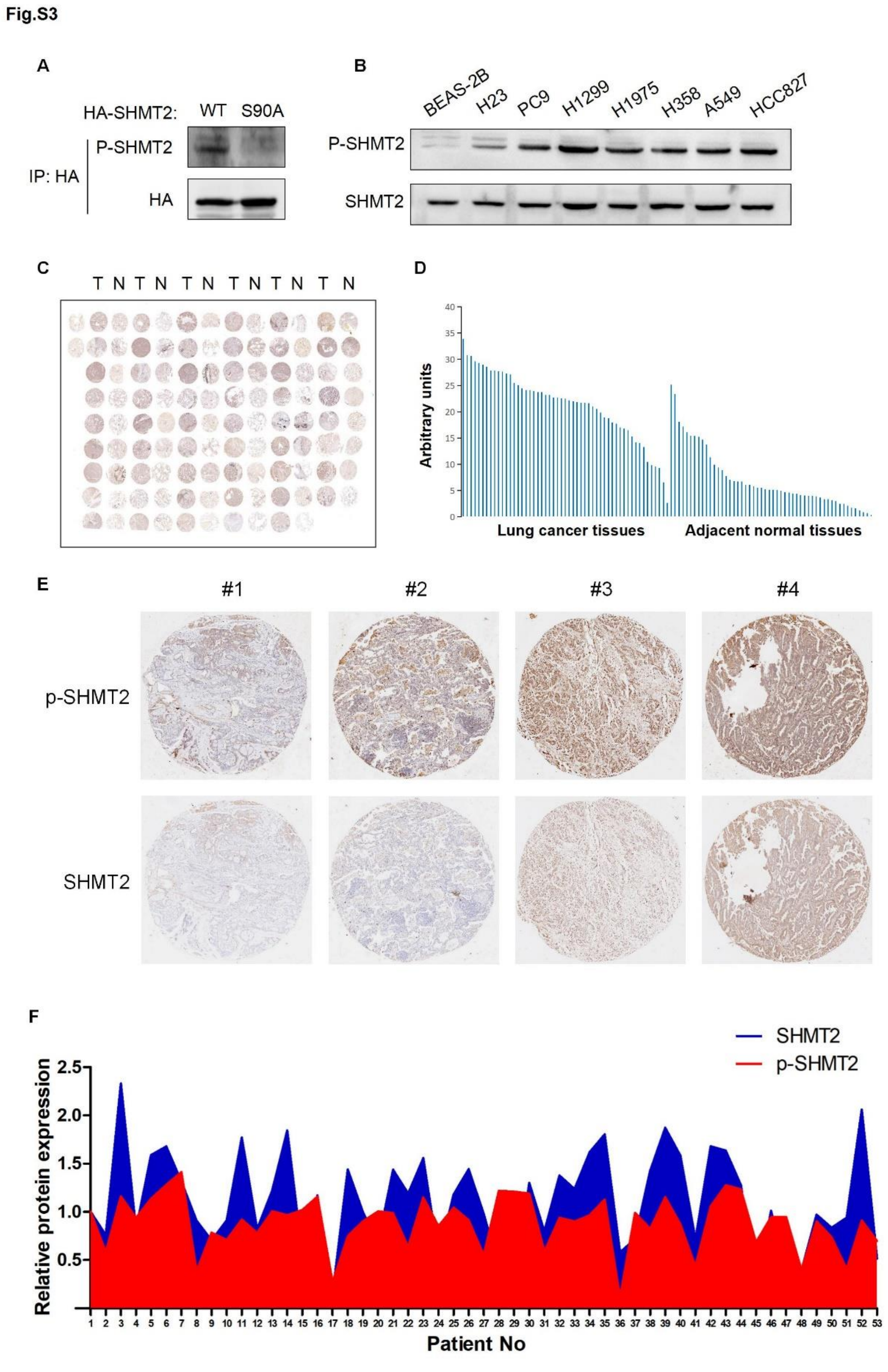

Figure S3. SHMT2-Ser90 phosphorylation is closely associated with the expression of SHMT2 in LUAD cells. (A) A549 cells were

transfected with HA-SHMT2 or HA-SHMT2-S90A. Immunoprecipitation was used to concentrate SHMT2 protein and SHMT2-pSer90 antibody (P-SHMT2) was used to detect protein phosphorylation. (B) The SHMT2-pSer90 antibody was used to detect the phosphorylation levels of SHMT2-Ser90 in human bronchial epithelial cells (BEAS-2B) and LUAD cells (H23, H1299, H358, H1975, A549, PC9, HCC827). Total SHMT2 expression was used as the loading control. (C) Microscopic evaluation of IHC staining of the LUAD tissue microarray with the SHMT2-pSer90 antibody. N: adjacent normal tissue; T: tumor tissue. (D) Quantification of the IHC staining shown in Figure S3C. (E) The representative images of the same tumor tissues with IHC staining shown in Figure 1J and S3C. (F) Evaluation of IHC staining of the LUAD tissue microarray with SHMT2 and SHMT2-pSer90 antibodies in Figure 1J and S3C.

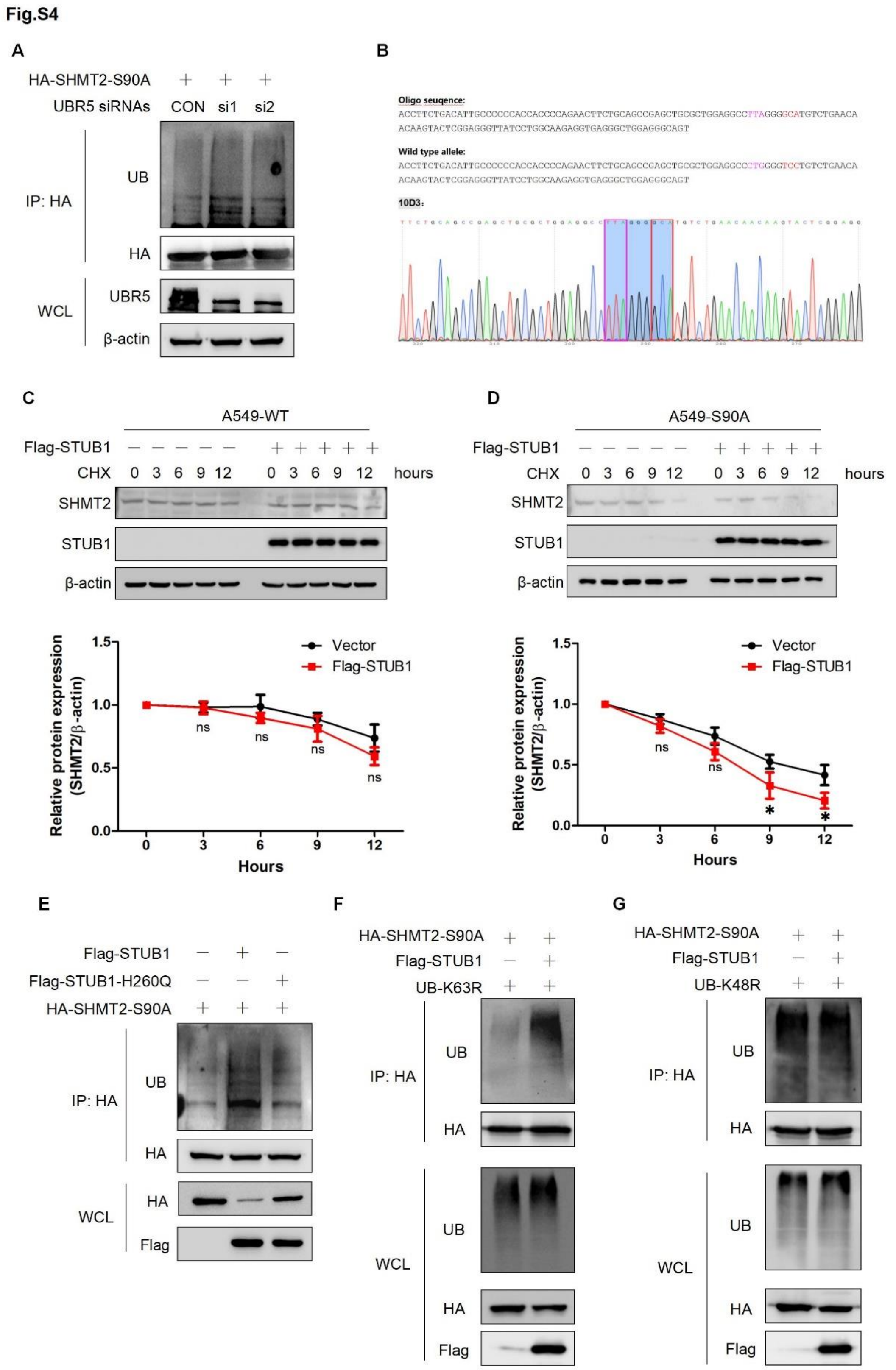

Figure S4. STUB1 regulates the ubiquitination and degradation of SHMT2-S90A. (**A**) A549 cells were co-transfected with

HA-SHMT2-S90A plasmid and negative control siRNA, or HA-SHMT2-S90A and UBR5 specific siRNAs. 48 hours later, The ubiquitination of SHMT2-S90A was detected using western blot. (**B**) schematic depiction of the sequencing results for SHMT2-S90A mutation in A549 cells. (**C and D**) A549-WT and A549-SHMT2-S90A cells were transfected with or without Flag-STUB1. CHX was used to treated the cells for indicated times and western blot was used to detect the protein expression using indicated antibodies (Top panels). Relative SHMT2 expression over β-actin was quantified. The data represent the average of three independent experiments (mean ± SD). ns, $P>0.05$; *, $P<0.05$ (Bottom panels). (**E**) A549 cells were transfected with indicated plasmids and immunoprecipitation combined with western blot were used to detected the ubiquitination. (**F and G**) The ubiquitin mutants K63R (F) and K48R (G) were co-transfected with the indicated plasmids. Immunoprecipitation combined with western blot were used to detect the protein expressions.

Fig.S5

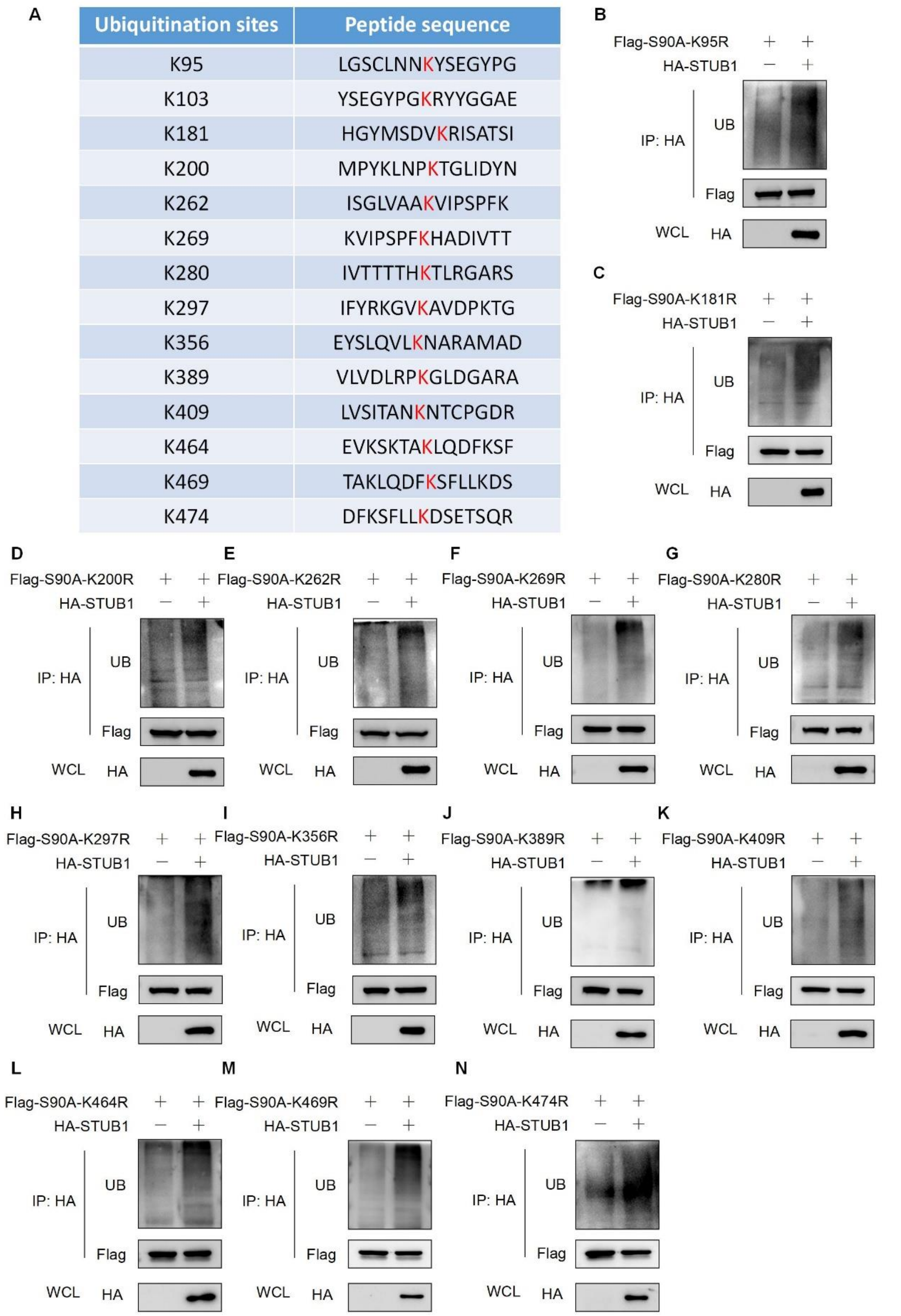

Ubiquitination sites	Peptide sequence
K95	LGSCLNNKYSEGYPG
K103	YSEGYPGKRYYGGAE
K181	HGYMSDVKRISATSI
K200	MPYKLNPKTGLIDYN
K262	ISGLVAAKVIPSPFK
K269	KVIPSPFKHADIVTT
K280	IVTTTTHKTLRGARS
K297	IFYRKGVKAVDPKTG
K356	EYSLQVLKNARAMAD
K389	VLVDLRPKGLDGARA
K409	LVSITANKNTCPGDR
K464	EVKSKTAKLQDFKSF
K469	TAKLQDFKSFLLKDS
K474	DFKSFLLKDSETSQR

Figure S5. Discovering the key ubiquitination site for the degradation of SHMT2-S90A. (A) The ubiquitination sites identified from

PhosphoSitePlus platform. (**B-N**) The indicated plasmids were transfected into A549 cells and Immunoprecipitation combined with western blot were used to detect the ubiquitination of SHMT2.

Fig.S6

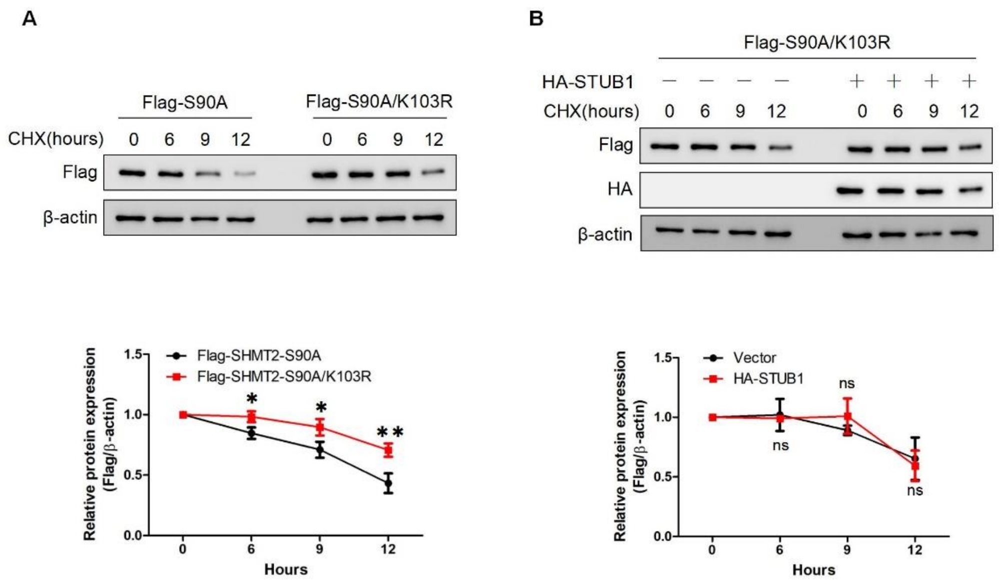

Figure S6. Lys103 is the key ubiquitination site in SHMT2-S90A regulated by STUB1. (**A**) Flag-SHMT2-S90A and Flag-SHMT2-S90A/K103R were transfected into A549 cells. CHX was used to treat the cells for the indicated times, and protein degradation was detected via western blotting (Top panel). Relative Flag-SHMT2 expression over β-actin was quantified. The data represent the average of three independent experiments (mean ± SD). *, P<0.05; **, P<0.01 (Bottom panel). (**B**) Flag-SHMT2-S90A/K103R plasmid with or without HA-STUB1 plasmid were transfected into A549 cells. CHX was used to

treat the cells for the indicated times, and protein degradation was detected via western blotting (Top panel). Relative Flag-SHMT2 expression over β-actin was quantified. The data represent the average of three independent experiments (mean ± SD). ns, $P>0.05$ (Bottom panel).

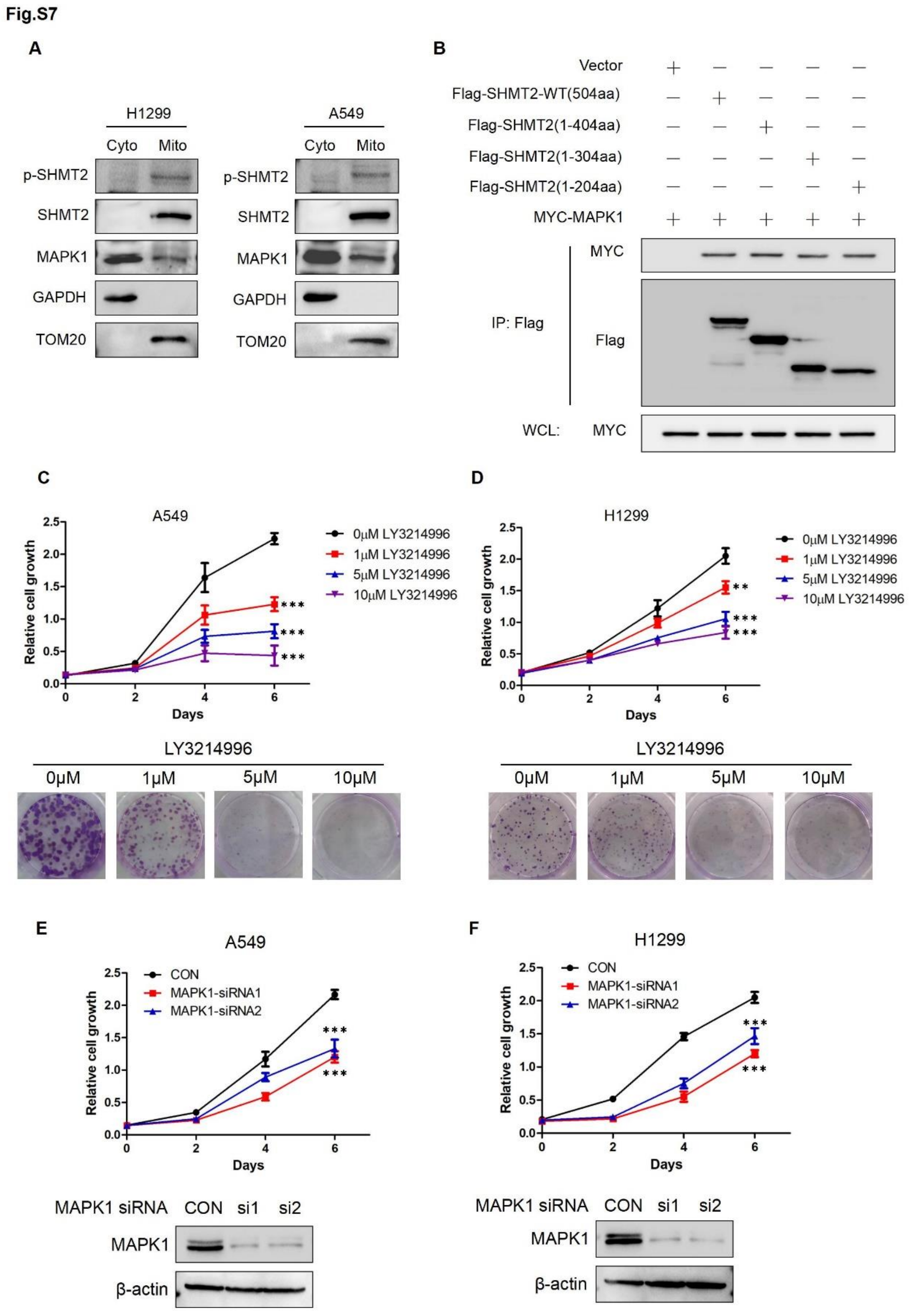

Figure S7. MAPK1 interacts with SHMT2 and regulates the proliferation of LUAD cells. (A) Mitochondria isolation was performed

in H1299 and A549 cells and western blotting was used to detect the protein expressions. Cyto: cytoplasm; Mito: mitochondria. **(B)** MYC-MAPK1 plasmid and Flag-SHMT2 plasmids with different truncations (WT: wild-type; aa: amino acid) were transfected into 293T cells. Immunoprecipitation combined with western blotting were used to detect the protein interactions. (**C and D**) LUAD cells (A549, H1299) were treated with different concentrations of LY3214996 followed by cell proliferation assay (Top panel) and colony formation assay (Bottom panel). Data represent the average of three independent experiments (mean±SD). **, P<0.01; ***, P<0.001. (**E and F**) LUAD cells (A549, H1299) were transfected with MAPK1 specific siRNAs followed by cell proliferation assay (Top panel). Data represent the average of three independent experiments (mean±SD). ***, P<0.001. The knockdown efficiency was detected using western blotting (Bottom panel).

Fig.S8

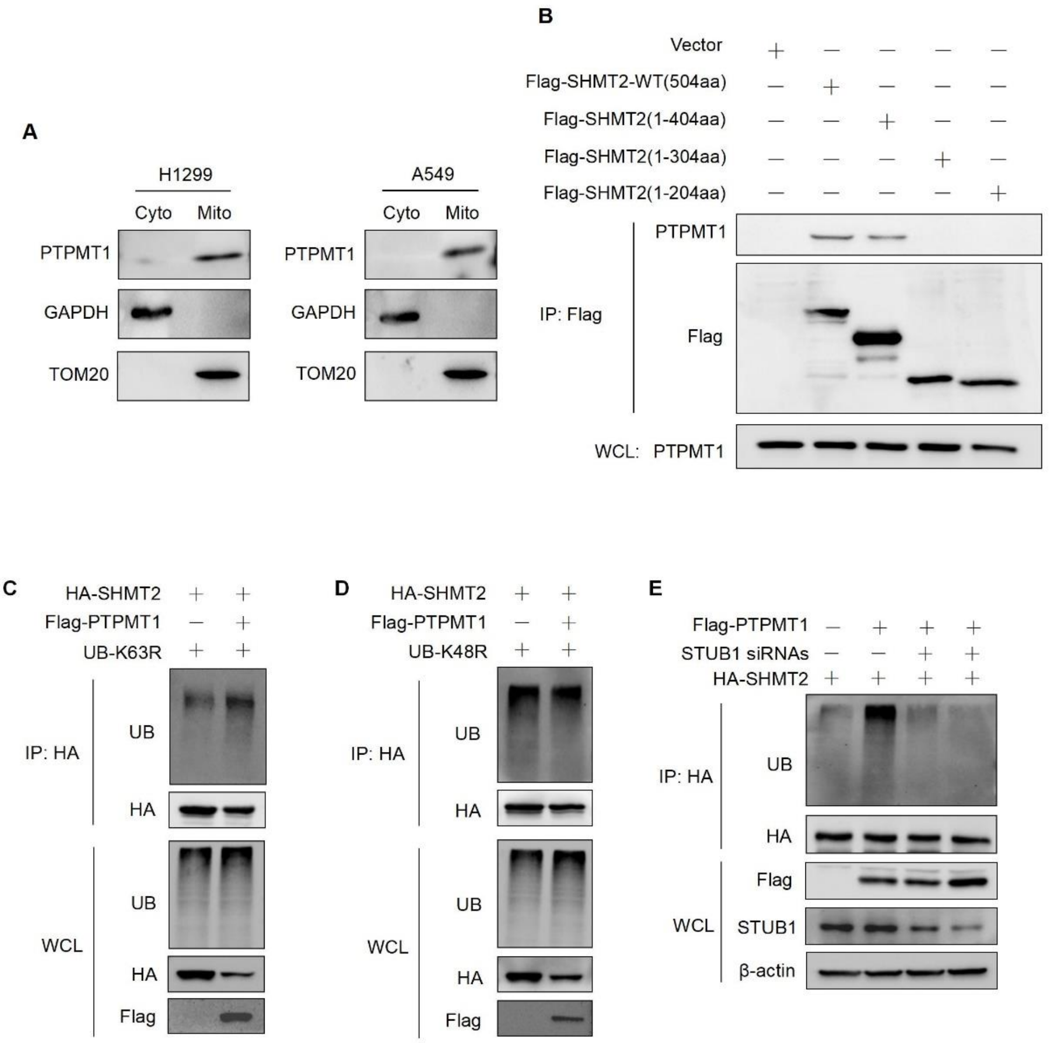

Figure S8. PTPMT1 interacts with SHMT2 and regulates its ubiquitination. (A) Mitochondria isolation was performed in H1299 and A549 cells and western blotting was used to detect the protein expressions. **(B)** The Flag-SHMT2 plasmids with different truncations (WT: wild-type; aa: amino acid) were transfected into A549 cells. Immunoprecipitation combined with western blotting were used to detect the protein interactions. (**C and D**) The K63R (C) and K48R (D) ubiquitin mutants were cotransfected with the indicated plasmids.

Immunoprecipitation combined with western blotting was used to detect protein expression. (**E**) A549 cells were transfected with the indicated plasmids and siRNAs. Immunoprecipitation was used to concentrate HA-SHMT2. Western blotting was used to detect protein expression.

Fig.S9

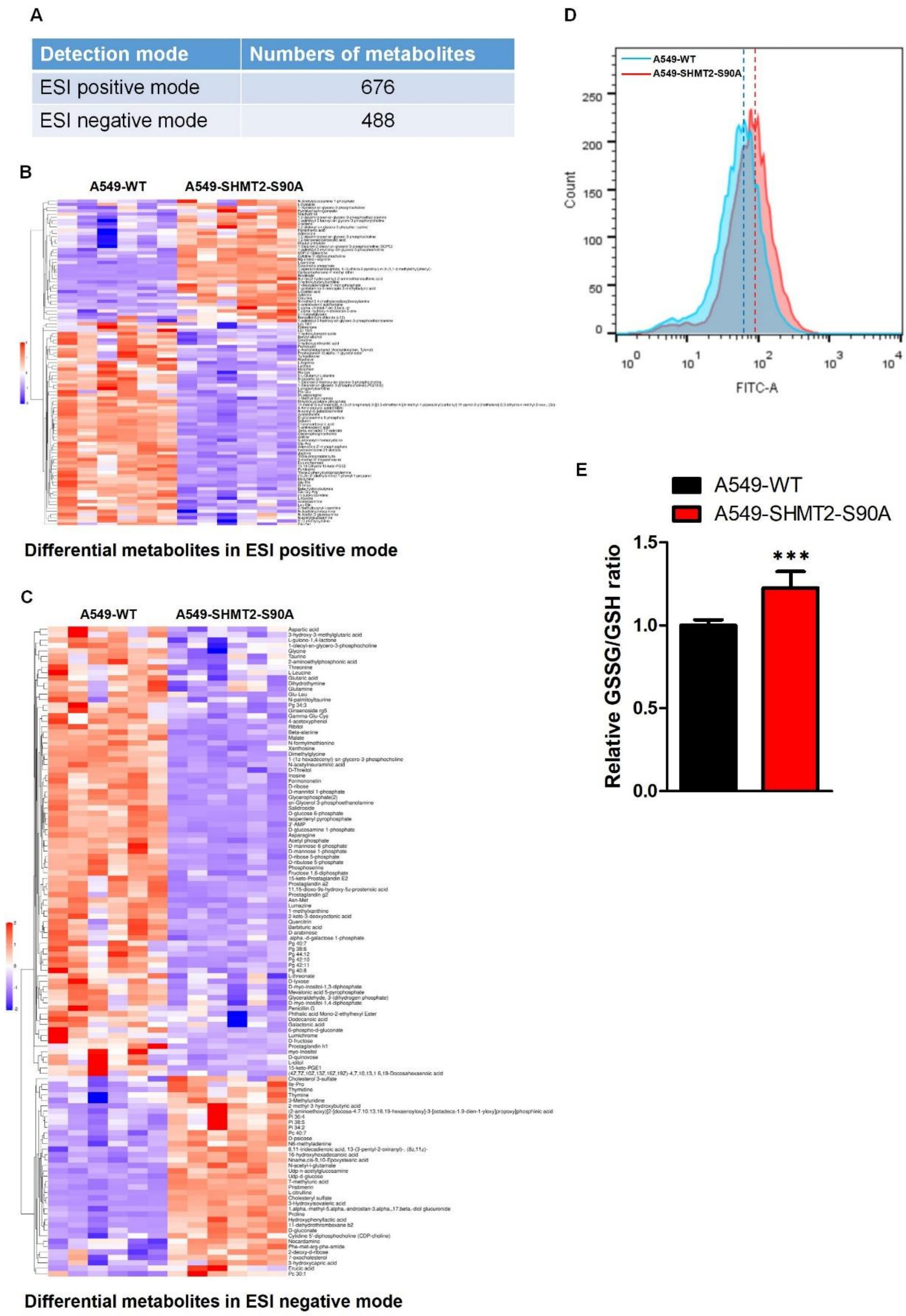

Figure S9. SHMT2-Ser90 phosphorylation regulates tumor metabolism. (**A**) The metabolites identified from Untargeted

Metabolomics Analysis. (**B and C**) The heat map of the metabolites identified from metabolomics analysis under ESI positive mode (B) and negative mode (C). (**D**) A549-WT and A549-SHMT2-S90A cells were treated with DCFH-DA and the ROS levels were detected using flow cytometer. (**E**) The GSSG/GSH ratio in A549-WT and A549-SHMT2-S90A cells were detected using GSH and GSSG assay kit. ***, $P<0.001$.

Fig.S10

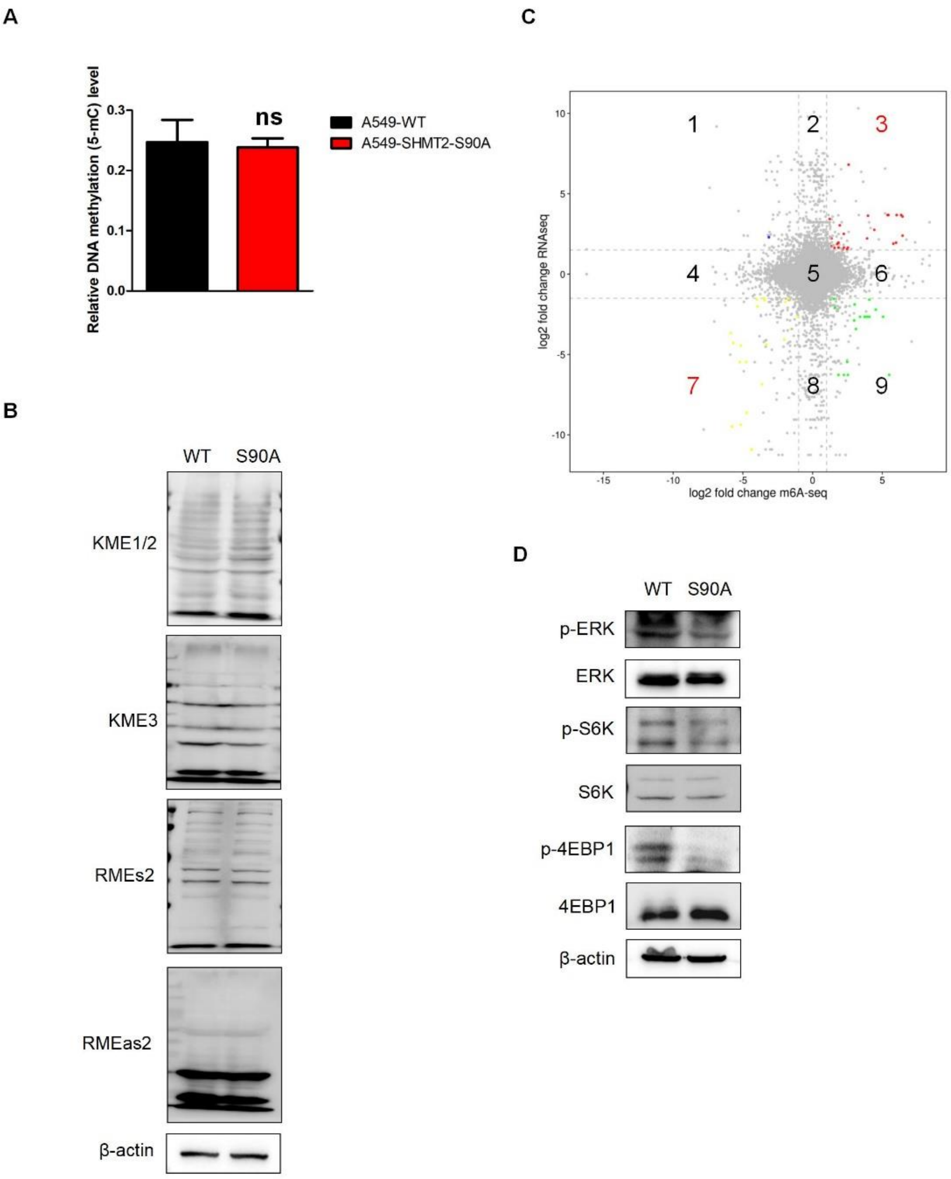

Figure S10. SHMT2-Ser90 phosphorylation regulates m6A methylation of global RNA. (**A**) The DNA methylation (5-mC) level in A549-WT and A549-SHMT2-S90A cells were detected using global DNA methylation (5-mC) kit. Data represent the average of three independent experiments (mean ± SD). ns, P>0.05. (**B**) The protein methylation in A549-WT and A549-SHMT2-S90A cells were detected by western blot using indicated antibodies. (**C**) Nine quadrant analysis of the

genes identified from combined analysis of MeRIP-Seq and RNA-Seq. The genes in Quadrant 3 and 7 indicated that the mRNA levels were positively correlated with the m6A methylation levels. (**D**) Western blot was used to detect the protein expression in A549-WT and A549-SHMT2-S90A cells.

Table information

Table S1. The Detailed Information of the screening Library.

Table S2. The phosphorylation sites of SHMT2 identified by MS.

Table S3. The binding proteins of SHMT2-WT and SHMT2-S90A identified by MS.

Table S4. The detailed information of untargeted metabolomics.

Table S5. The differential m6A peaks identified via MeRIP-Seq

Table S6. Combined analysis of MeRIP-Seq and RNA-Seq.

Table S7. Clinical pathological materials of Lung adenocarcinoma tissue microarray (ALC-1904).

Table S8. Clinical pathological materials of Lung adenocarcinoma tissue microarray (HLugA180Su11).
